# Multiphoton Bleaching of Red Fluorescent Proteins and the Ways to Reduce It

**DOI:** 10.3390/ijms23020770

**Published:** 2022-01-11

**Authors:** Mikhail Drobizhev, Rosana S. Molina, Jacob Franklin

**Affiliations:** 1 Department of Microbiology and Cell Biology, Montana State University, Bozeman, MT 59717, USA; zerlinia@gmail.com; 2 Vidrio Technologies LLC, 19955 Highland Vista Drive Suite 150, Ashburn, VA 20147, USA; jacob@vidriotech.com

**Keywords:** red fluorescent proteins, mCherry, mPlum, tdTomato, jREX-GECO1, multiphoton absorption, two-photon laser microscopy, multiphoton bleaching, photoionization, electron photodetachment

## Abstract

Red fluorescent proteins and biosensors built upon them are potentially beneficial for two-photon laser microscopy (TPLM) because they can image deeper layers of tissue, compared to green fluorescent proteins. However, some publications report on their very fast photobleaching, especially upon excitation at 750–800 nm. Here we study the multiphoton bleaching properties of mCherry, mPlum, tdTomato, and jREX-GECO1, measuring power dependences of photobleaching rates *K* at different excitation wavelengths across the whole two-photon absorption spectrum. Although all these proteins contain the chromophore with the same chemical structure, the mechanisms of their multiphoton bleaching are different. The number of photons required to initiate a photochemical reaction varies, depending on wavelength and power, from 2 (all four proteins) to 3 (jREX-GECO1) to 4 (mCherry, mPlum, tdTomato), and even up to 8 (tdTomato). We found that at sufficiently low excitation power *P*, the rate *K* often follows a quadratic power dependence, that turns into higher order dependence (*K~P*^α^ with α > 2) when the power surpasses a particular threshold P*. An optimum intensity for TPLM is close to the P*, because it provides the highest signal-to-background ratio and any further reduction of laser intensity would not improve the fluorescence/bleaching rate ratio. Additionally, one should avoid using wavelengths shorter than a particular threshold to avoid fast bleaching due to multiphoton ionization.

## 1. Introduction

Red fluorescent proteins (red FPs, RFPs) are genetically encoded molecular probes that find use in fluorescence microscopy of biological tissues, such as the brain [1,2,3,4,5]. Their red-shifted absorption and fluorescence provide structural and dynamic information from deeper layers of tissue, compared to their green counterparts, because red light penetrates better in scattering media. RFPs are particularly attractive for use in two-photon laser microscopy (TPLM) [6,7,8,9], where two near-infrared photons can effectively excite the RFP chromophore [10]. Two-photon excitation (2PE) spectra of RFPs fall in the range from 700 to 1200 nm [10,11] that matches the so-called tissue transparency window [12]. They show two main peaks in this range. The first one, at 1050–1200 nm, typically has moderate two-photon absorption (2PA) cross section, *σ*_2_ = 10–100 GM (1 GM = 10^−50^ cm^4^ s). The second one, at 700–780 nm, is usually stronger, with *σ*_2_ reaching 100–300 GM in some RFPs. This latter region corresponds to a higher energy, S_0_ → S_m_, transition that is also present in one-photon absorption spectra as a weak peak near 350–380 nm. When excited with two photons, it gains its intensity due to the nonlinear optical effect of pre-resonance enhancement that occurs when the laser photon energy approaches the lowest S_0_ → S_1_ transition energy from below, but still does not populate it [13,14]. Therefore, excitation at 700–780 nm still corresponds to a simultaneous two-photon absorption with all advantages offered by TPLM, including high spatial (3D) resolution, low out-of-focus damage of tissue, and low autofluorescence background. Given the availability of commercial femtosecond Ti:sapphire lasers, with the high output power and stable operation in this spectral region and the possibility of dual-color two-photon imaging with simultaneous excitation of red and blue FPs [15], excitation at 700–780 nm provides a very attractive opportunity for TPLM with RFPs. However, researchers quickly discovered that RFPs bleach extremely fast when using femtosecond lasers operating in this wavelength range, with typical pulse-peak photon fluxes of 10^27^–10^29^ photon/cm^2^/s at the sample.

There are a number of reports investigating multiphoton bleaching of fluorescent dyes and FPs, but the molecular mechanisms remain almost completely unexplored. In their seminal work, Patterson and Piston reported on multiphoton bleaching of fluorescein dextran, aminocoumarin dextran, NADH, and Indo-1 [16]. Using a femtosecond 710 nm excitation, they found that although the initial fluorescence intensity increased quadratically with laser power *P*, the photobleaching rate *K* increased much faster (i.e., according to a power law, *K~P^α^* with the exponents *α* ranging from 3 to 5). This super-quadratic dependence suggested involvement of multiphoton processes with number of photons *n* > 2. It is clear that after direct two-photon excitation, that requires very high photon flux, a large organic molecule can readily undergo several stepwise one-photon absorption events, through a ladder of electronic/vibrational excited states, that make a quasi-continuum above the S_1_ level. The most general photochemical outcome of this process is multiphoton ionization or electron detachment (for anionic chromophores) [17]. For example, fluorescein dextran required absorption of a total of three photons (power exponent 3.1 ± 0.1) for photobleaching. Photodetachment energy of fluorescein lies between 5.1 and 6.3 eV (depending on protonation state) [18]. For the 1.75 eV photon energy (used in the Patterson and Piston experiment) the above numbers translate into the number of photons, *n* ~3, thus supporting the photodetachment mechanism. Photobleaching of aminocoumarin requires five photons (power exponent 5.1 ± 0.2 at 710 nm) [16]. Given photoionization energy of aminocoumarin of 8.05 eV [19], a minimum of five photons are required to ionize this molecule, which supports our assumption that the photobleaching is caused by multiphoton ionization.

Marchant and co-authors [20] demonstrated that the wild-type red FP, DsRed, bleaches in CHO cells according to a third power law (*α* = 2.98 ± 0.10) when excited at 760 nm, in the range of average power densities 5–40 μW/μm^2^ (approximately corresponding to peak photon fluxes of *I*_0_ = 3 × 10^26^–2.5 × 10^27^ photon/cm^2^/s). This suggests a three-photon initiated process. The authors also found that, upon excitation at 950 nm, DsRed bleaches much slower than at 750 nm excitation, with the laser power adjusted to produce similar initial fluorescence signals at these two wavelengths. Later, Robinson and Marchant [21] extended this study to several DsRed mutants expressed in HEK-293 cells, and have shown that all of them bleach much faster at 750 nm compared to 950 nm. The power exponents observed for bleaching at 750 mn varied from 2.69 (for DsRed2) to 3.05 (for DsRed [N42Q]). A similar trend of slowing down the bleaching rate upon shifting excitation of DsRed2 to longer wavelengths, from 760 to 880 and further to 1100 nm, was found in [22]. Higher than quadratic power dependence (*α* = 2.9 ± 0.10) was also observed in another DsRed mutant, tdRFP, expressed in T cells and excited at 1100 nm with *I*_0_ = (2–3.5) × 10^29^ photon/cm^2^/s [23]. Interestingly, the same excitation fluxes resulted in much faster bleaching of enhanced green fluorescent protein (EGFP) at 850 nm, with *α* = 2.57 ± 0.25. Other groups also reported a super-quadratic dependence of photobleaching for EGFP and cyan FP (CFP) [24,25,26,27]. Kalies et al. correlated the power exponent of the EGFP bleaching rate with the multiphoton electron photodetachment from the chromophore or a nearby tryptophan residue [26].

Drobizhev and co-authors characterized multiphoton bleaching of monomeric RFPs, mFruits (mCherry, mPlum, and mStrawberry) and DsRed2 in buffer solution and *E*. *coli* cells, with the emphasis on possible photochemical mechanisms [28]. The rate of the first step of a chain of photochemical reactions leading to photobleaching was measured as a function of laser intensity at 790 nm. The DsRed2 rate followed a power law with *α* = 2.84 ± 0.01, at *I*_0_ = (0.3–1.1) × 10^29^ photon/cm^2^/s, in agreement with previous observations [20,21]. In DsRed2, photobleaching occurs through the three-photon absorption process with a slight saturation of the one-photon S_m_ → S_n_ transition. Vertical detachment energy (VDE) of the anionic chromophore inside DsRed2 lies at ~7.24 eV [28], suggesting that at least five photons (of 790 nm) are needed to reach this threshold. A three-photon-induced electron transfer reaction starting from the bound S_n_ state of DsRed2 is probably a first step of phototransformation [28]. mFruits bleach according to a power law with *α* = 3.2–3.5, suggesting a four-photon mechanism with strong saturation of one or two one-photon transitions, following initial two-photon absorption step. Electron photodetachment energies of mFruits are found in the range 5.52 (mCherry)–5.61 eV (mPlum) [28]. Absorption of four photons (at 790 nm) can easily provide enough energy to overcome this threshold and, therefore, the four-photon photodetachment is suggested as a first step of photobleaching in mFruits [28]. The chromophore environment of DsRed2 contains more positive amino acid residues compared to mFruits, and this can be a reason of its higher photodetachment energy.

Since the fluorescence signal increases slower (quadratically), with the laser peak intensity compared to photobleaching rate, any reduction of the laser pulse-peak intensity should be beneficial until the moment where fluorescence signal becomes undetectable against the noise. Following this idea, several approaches to reduce photobleaching were suggested. Drummond et al. proposed to (1) block the laser with the programmed Pockels cell during the “flyback” time (when fluorescence is not collected but the laser still irradiates the sample); and (2) increase the sensitivity of detection by using an additional widefield detector in the transmission position, which made it possible to use less excitation power [25]. The use of passive pulse splitters, providing less peak intensity but higher repetition rate, resulted in faster reduction of photobleaching relatively to fluorescence signal [29]. On the other hand, if multiphoton absorption involves triplet manifold, lower pulse repetition rate can be beneficial [30]. Phase modulation or dispersion compensation of femtosecond pulses also helped reducing multiphoton bleaching [27,31,32,33]. Although these methods provide some improvement on the fluorescence/bleaching ratio, they require technical upgrades of commercial 2P microscopes.

Here we aim at finding simple solutions to the problem of rapid multiphoton bleaching in terms of optimization of laser intensity and excitation wavelength. They require the (phenomenological) knowledge of photochemical mechanisms and their dependence on laser intensity and wavelength. First, one can expect that at small laser intensities the bleaching rate should show a quadratic dependence on intensity, with the rate equal to that observed with one-photon excitation (with the 2P and 1P excitation rates being equal). A transition between quadratic and higher-order dependence defines a particular threshold for laser power, P*. An optimum intensity for TPLM should be chosen close to the P* value because it provides the highest signal-to-background ratio (SBR), and any further reduction of intensity would not improve the fluorescence/bleaching ratio. A particular value of threshold intensity is a function of wavelength because the corresponding laser and molecular parameters depend on wavelength. Second, if the bleaching is due to multiphoton ionization (electron detachment) with *n* photons involved (like in mFruits), one can look for a threshold photon energy (wavelength) where this process in not allowed energetically: *nh*ν < *VDE*.

We check these ideas for a series of RFPs, including popular monomeric variants mCherry [1] and mPlum [34], one of the two-photon brightest RFP, tdTomato [1,10], and a new red Ca^2+^ sensor with the long Stokes shift in the Ca^2+^-bound state, jREX-GECO1 [35]. Compared to the previous work [28], we study photobleaching rates in a broader range of excitation intensities and at different excitation wavelengths across the 2PE spectrum. We found some important threshold values for the laser photon fluxes and wavelengths for these RFPs and show that in some cases one can predict these values if the molecular and laser parameters are known.

## 2. Results

### 2.1. Physical Model of Multiphoton Bleaching in a Thin Colony of Cells Containing Fluorescing Molecules

We use a physical model of multiphoton bleaching that was previously described in [28]. Here we briefly present its main points and summarize its results.

**Figure 1:**
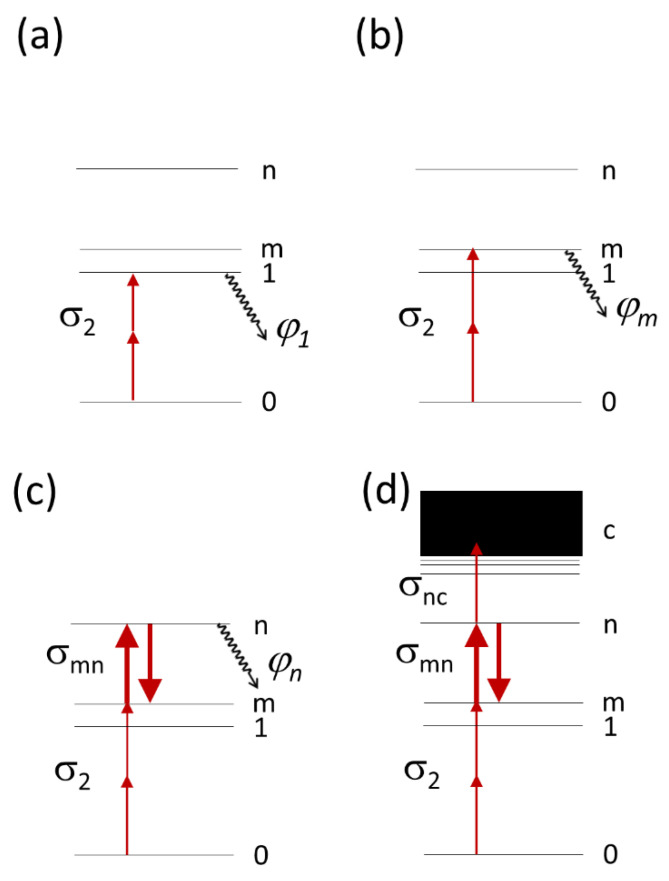
Energy level diagrams for some multiphoton induced photobleaching processes. Panels (**a**,**b**) correspond to two-photon bleaching following initial two-photon absorption with the cross section σ_2_. Panel (**c**) corresponds to a three-photon bleaching where initial 2P excitation to level m (m can be equal to 1) is followed by a one-photon transition m → n with the 1PA cross section, *σ*_mn_. The bold arrows connecting m and n states designate strong absorption (up) and stimulated emission (down) resulting in possible saturation of the m → n transition. Panel (**d**) shows a four-photon bleaching process, where, after three-photon excitation to level n, an electron leaves a molecule with photoionization (photodetachment) cross section *σ*_nc_, and transits into a continuum of states (**c**).

After absorption of *n* photons, a molecule can undergo a chain of chemical transformations leading to eventual bleaching of fluorophore,

(1) $$A^{*}⇄B^{\left(*\right)}\to C\to$$

where *A* is the concentrations of the initial form, and *B*, *C*, etc. are the concentrations of intermediate forms. The asterisk denotes an electronically excited state. This process results in a complex, non-monoexponential decay kinetics of the initial state *A*. For simplicity, here we measure the initial slope of the fluorescence decay curve *F*(*t*) normalized to 1 at *t* = 0 (method of initial rates of chemical reaction). This slope provides the observable rate *K* that, in turn, is related to molecular rate of the first reaction step, *k*_1_, corresponding to the A* → *B*^(^*^)^ transition. To express *k*_1_ through molecular and laser parameters, we consider dynamics of populations in several different energy level diagrams displayed in Figure 1.

The diagram (a) represents a low intensity (lower than a certain threshold) two-photon excitation of molecule to the first excited electronic level S_1_, from which it can start a transformation with quantum yield *φ*_1_. The rate *k*_1_ depends quadratically on laser power in this regime. This process should obey the well-known Kasha’s rule that states that independently of the mode of excitation (i.e., one- or two-photon), the quantum yield *φ*_1_ should be the same.

The diagram (b) corresponds to a low-intensity two-photon excitation to a higher electronic level S_m_. After such excitation, a molecule can either relax to the S_1_ state with subsequent transformation to *B* as in case (1) (standard Kasha’s pathway), or proceed to *B* with different quantum yield, *φ*_m_ (violating Kasha’s rule). The latter corresponds to a situation when the A* → *B*^(^*^)^ transition efficiency depends on the excess excitation energy in the A* state relatively to the lowest level of the S_1_ state. One can think that this can only happen if the photochemical quantum yield *φ*_m_ ~1, because the photochemical rate should be on the picosecond scale (i.e., competing with intramolecular vibrational relaxation). However, the possibility of the reverse reaction B* → A* or even B* → *B* → *A* removes that stringent requirement and the *φ*_m_ value is still allowed to be much less than 1. In other words, a violation of Kasha’s rule is related to a presence of a second specie, B* that is dynamically coupled to A*.

The third diagram (c) represents the three-photon process where the initial simultaneous two-photon excitation (0 → m) is followed by a third photon, absorbed linearly through the m → n transition. The quantum yield of the reaction, initiated from the excited state n, *φ*_n_, can be different from *φ*_1_ and from *φ*_m_, and the same considerations as applied to *φ*_m_ can be used here. This three-photon (2 + 1)-induced process occurs at moderate laser intensities, typical for TPLM. Even with moderately high intensities needed for the robust two-photon excitation (10^28^–10^29^ photons/sm^2^/s), once a molecule is in the m state, the probability of the m → n transition (bold up arrow in Figure 1c) can be very high. This will result in partial saturation of transition. In fact, for typical one-photon cross sections, *σ*_mn_ ~10^−16^ cm^2^, the probability of absorption during the pulse duration (10^−13^ s) becomes ~0.1–1, and the stimulated emission must be considered as well (bold down arrow in Figure 1c). As a result, the intensity dependence of the rate will show a power dependence with an exponent *α* between 2 and 3. The last diagram (d) demonstrates the process of four-photon (2 + 1 + 1)-induced bleaching involving initial two-photon absorption followed by sequential one-photon absorptions of two additional photons, eventually bringing the electron to an ionization continuum that lies above the ionization energy (electron detachment energy for anionic chromophores). The last step transition n → c typically has small cross sections, *σ*_nc_ ~10^−20^–10^−19^ cm^2^ ([17] and the data below).

Our estimation shows that with the intensities used here, it is difficult to saturate this transition. In the case of partial saturation of the m → n transition, the power exponent of the bleaching rate will be between 3 and 4.

The solution of the rate equations corresponding to cases (a)–(d) at a particular photon flux for “local” decay rates was given in [28]. In our experiment, we irradiate *E. coli* cells expressing a particular RFP in a 2PLM setup and measure the temporal decay of fluorescence signal. In the description of the observed multiphoton bleaching kinetics, we neglect the diffusion of FPs in and out of laser focal area during bleaching time because the laser beam diameter in our experiment is much larger than the *E. coli* cell size. However, because of the spatial distribution of intensity, different chromophore molecules will see different photon fluxes. To obtain the observed (average) rates we had to integrate the local rates over space and time. It is important that the colony thickness *l* in our experiments is comparable to the Rayleigh length *z_R_* of the laser intensity distribution. Therefore, we do not use a “thick sample” approximation and the integration results depend on geometrical parameter equal to the ratio *η* = *l*/2*z_R_*.

To proceed with integration, we describe the spatial and temporal distribution of laser intensity near the focal spot by a Gaussian-Lorentzian function in cylindrical coordinates,

(2) $$\begin{array}{c} I\left(r,z,\tau \right)=I_{0}\frac{w_{0}^{2}}{w{\left(z\right)}^{2}}exp\left(-\frac{2r^{2}}{w{\left(z\right)}^{2}}\right),\;-\Delta \tau /2\leq \tau <\Delta \tau /2;\\ I\left(r,z,\tau \right)=0,\;otherwise, \end{array}$$

where *w*(*z*) is the beam waist radius at distance *z* from the focal plane (in cm^2^), *τ* is the time lapse within the pulse (in s), *I*_0_ = *I* (0,0,0) is the peak photon density (in photons/cm^2^ s) in focal spot. For simplicity, we consider the laser pulse temporal profile as rectangular with a full width of ∆*τ*. Integration of (2) over time and cross-sectional area at focal plane *z* = 0 (*w* = *w*_0_) relates the peak photon density *I*_0_ to the average laser power *P* (in W), measured in experiment:

(3) $$P=\frac{\pi }{2}fh\nu \Delta \tau w_{0}^{2}I_{0},$$

where *f* is the pulse repetition rate, *h* is the Planck constant, *ν* is the photon frequency (in Hz).

We now calculate the initial decay rate of the observed fluorescence signal normalized to initial signal (*F*(0)) as follows:

(4) $$K=-\frac{1}{F\left(0\right)}{\frac{dF}{dt}|}_{t=0}=-\frac{\int dV\int_{pulse}^{}d\tau I^{2}\left(r,z,t\right){\frac{\partial n\left(r,z,t\right)}{\partial t}|}_{t=0}}{n\left(0\right)\int dV\int_{pulse}^{}d\tau I^{2}\left(r,z,t\right)},$$

where *t* is the measurement time, *dV* is the element of volume *dV* = 2π*rdrdz*, *n*(*r,z,t*) is the time-dependent local concentration of unbleached molecules.

In general, the multiphoton rate can contain different terms corresponding to processes with different orders that potentially compete with each other:

$$K=K^{\left(2\right)}+K^{\left(3\right)}+K^{\left(4\right)}+\ldots$$

We now provide the analytical results, corresponding to individual terms in the above equation without and with partial saturation of some of the intermediate transitions. In the case (a) of Figure 1, the observed rate of two-photon bleaching process reads

(5) $$K^{\left(2\right)}=\frac{1}{\pi^{2}}\sigma_{2}\varphi_{1}\frac{\Gamma_{3}\left(\eta \right)}{\Delta \tau f{\left(h\nu \right)}^{2}w_{0}^{4}}P^{2},$$

where all Γ_i_(*η*) functions, i = 2, 3, 4, 5, appearing here and throughout depend only on the ratio *η* = *l*/2*z_R_*. They are presented in Supplementary Info. In the case (b) of Figure 1, one can use the same expression for *K*^(2)^ where *φ*_1_ is replaced by *φ*_m_.

In the case of three-photon initiated process (c) without saturation one obtains,

(6) $$K^{\left(3\right)}=\frac{4}{5\pi^{3}}\sigma_{2}\sigma_{\mathrm{mn}}\varphi_{n}\frac{\Gamma_{4}\left(\eta \right)}{\Delta \tau f^{2}{\left(h\nu \right)}^{3}w_{0}^{6}}P^{3}.$$

In the case of slight saturation of the m → n transition (i.e., when the observed power exponent is slightly less than 3), one can use the following fitting function:

(7) $$K^{\left(3\right)}=\frac{1}{\pi^{2}}\frac{\sigma_{2}\varphi_{n}}{\Delta \tau f{\left(h\nu \right)}^{2}w_{0}^{4}}\left[\frac{1}{5}\Gamma_{4}\left(\eta \right)\frac{P}{P_{s}}-\frac{1}{18}\Gamma_{5}\left(\eta \right){\left(\frac{P}{P_{s}}\right)}^{2}\;\right]P^{2},$$

where

(8) $$P_{s}=\frac{\pi }{4}\frac{fh\nu w_{0}^{2}}{\sigma_{\mathrm{mn}}}$$

is the saturation power (in W).

In the case of four-photon initiated process without saturation, Figure 1c, the observed rate reads

(9) $$K^{\left(4\right)}=\frac{4}{9\pi^{4}}\sigma_{2}\sigma_{\mathrm{mn}}\sigma_{\mathrm{nc}}\frac{\Gamma_{5}\left(\eta \right)}{\Delta \tau f^{3}{\left(h\nu \right)}^{4}w_{0}^{8}}P^{4}.$$

For the strong saturation of the m → n transition, (i.e., when the observed power exponent is slightly larger than 3), one can use an approximation:

(10) $$K^{\left(4\right)}=\frac{1}{4\pi }\frac{\sigma_{2}\sigma_{\mathrm{nc}}}{\sigma_{\mathrm{mn}}^{2}}\frac{1}{\Delta \tau f{\left(h\nu \right)}^{2}w_{0}^{4}}\left[\frac{1}{10}\Gamma_{4}\left(\eta \right){\left(\frac{P}{P_{s}}\right)}^{2}-\frac{1}{4}\Gamma_{3}\left(\eta \right)\left(\frac{P}{P_{s}}\right)+\frac{1}{3}\Gamma_{2}\left(\eta \right)\;\right]P.$$

### 2.2. Multiphoton Bleaching of mCherry in E. coli Colonies

**Figure 2:**
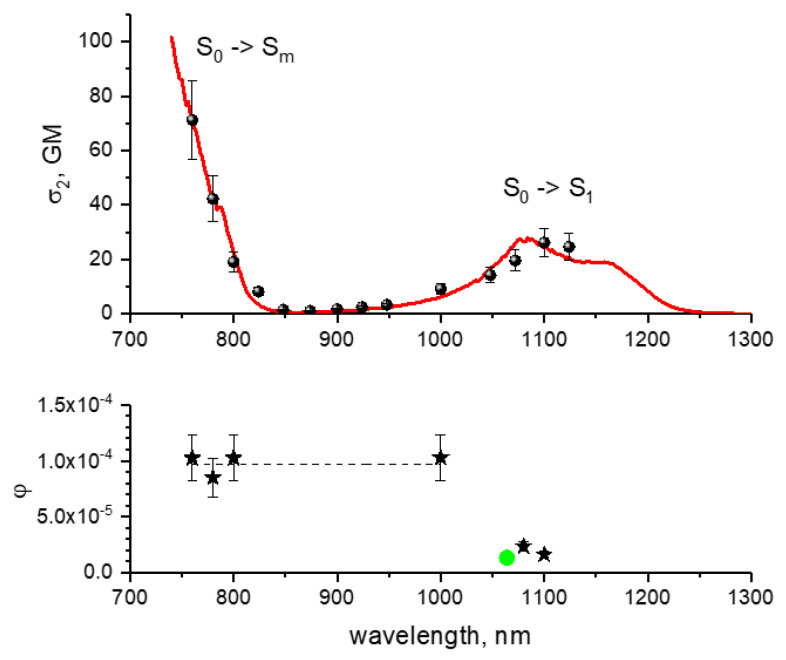
**Top**: Two-photon absorption spectrum of mCherry in purified buffer solution at pH8, (red line) [11] and two-photon excitation spectrum of mCherry measured in *E. coli* cells (symbols) (normalized to the 2PA cross section of solution spectrum at 760 nm). **Bottom**: Quantum yield of the first step of photobleaching process initiated by absorption of two photons as a function of excitation wavelength (black asterisks) for mCherry in *E. coli*. Quantum yield of the first step of one-photon induced photobleaching process upon laser excitation at 532 nm [36] is shown by a green circle at double wavelength (1064 nm).

We first measure the relative corrected 2PE spectrum of mCherry in *E. coli* cells from the same colony that we use in bleaching experiments and compare it to the spectrum previously observed in buffer solution [11]. Both spectra are shown in Figure 2 (top), with the spectrum in *E. coli* normalized to the 2PA cross section of mCherry in solution at 760 nm. The two spectra overlap quite well, confirming that the *E. coli* colony does express mCherry. The first electronic transition (S_0_ → S_1_) occurs at 1000–1200 nm, and the higher-energy and stronger electronic transition (S_0_ → S_m_) emerges at wavelengths shorter than 800 nm.

**Figure 3:**
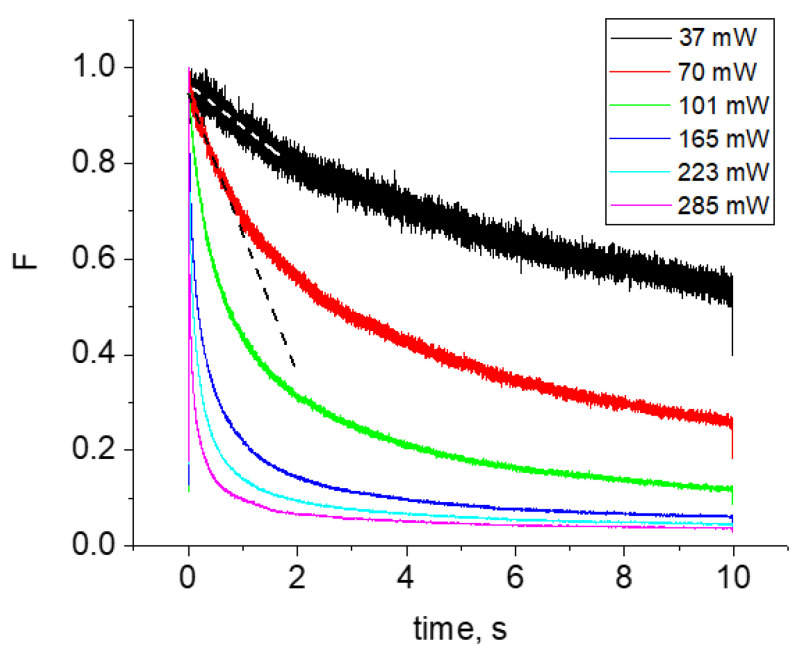
Fluorescence decay kinetic curves obtained at λ = 800 nm at different power values at the sample, shown in the inset. The kinetics are normalized to the *F*(0) = 1 values. The dashed lines illustrate the derivatives of the decay curves at *t* = 0, corresponding to 37 and 70 mW.

Next, we measured the power dependences of the bleaching rates of mCherry expressed in *E. coli* cells in a broad range of powers and at several wavelengths across the 2PA spectrum. The bleaching wavelengths correspond to the positions of asterisks in the bottom panel of Figure 2. They fall in both the S_0_ → S_1_ and S_0_ → S_m_ absorption bands. Figure 3 shows an example of the six fluorescence decay curves obtained at 800 nm upon bleaching at six different points within one colony with different laser powers *P*. For two of them, corresponding to *P* = 37 and 70 mW, we show here the linear fits of the initial decay stage (dashed lines). The *K* values were determined as the negative slopes of these lines (cf. Equation (4)). A very sharp increase of the decay rate is observed with the increase of intensity.

**Figure 4:**
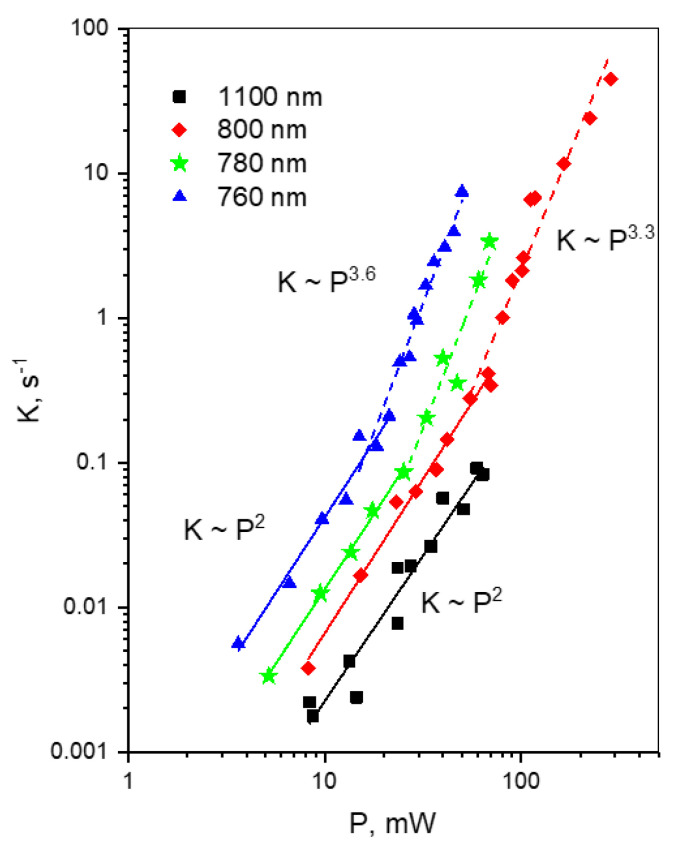
Power dependences of the initial bleaching rate of mCherry at different excitation wavelengths (see legend in the inset), presented in double logarithmic scale.

Figure 4 shows the series of power dependences of the rate K obtained at different excitation wavelengths. We observe several interesting trends.

(1) For each wavelength, there is a range of power values where the bleaching rate depends quadratically on power. Although this behavior is natural and reflects the fundamental bleaching limit corresponding to photochemical reaction starting just after absorption of two photons, to the best of our knowledge there are no publications where quadratic law was shown for any FP. After reaching a certain threshold, the dependence switches to super-quadratic, with α = 3.3–3.6. Note that previously the bleaching of mCherry was measured at 790 nm at similar excitation conditions (same objective lens, similar colony thickness and pulse duration) with the laser power of 40–150 mW [28], and the *K~P*^3.4^ dependence was observed. As one can see from Figure 4, these power levels were higher than the threshold (at 780–800 nm) and therefore the quadratic part was missed there.
(2) The power threshold P* for the super-quadratic transition is observed for wavelengths 760–800 nm, and it shifts to lower power values as the wavelengths becomes shorter.
(3) We did not observe a transition from quadratic to super quadratic dependence for wavelengths of 1100 (black squares) and 1000 nm (data not shown), at least for highest available power levels. Given the vertical electron detachment energy of mCherry is 5.52 eV [28], one can predict that the absorption of four photons with energy less than 1.57 eV (>900 nm) would not suffice to photodetach an electron. As a result, we would not anticipate the transition from *P*^2^ to *P*^3.5^ dependence for wavelengths longer than 900 nm. This imposes a critical wavelength for two-photon excitation of mCherry, λ = 900 nm, suggesting that using the lowest, S_0_ → S_1_ transition at 1000–1100 nm should result in slower (quadratic) multiphoton bleaching even at high powers.

To analyze the quadratic segments of the power dependences at different wavelengths (Figure 4), we plot the data obtained in the low power regime in the *K* vs. *P*^2^ coordinates and fit them to the straight line to obtain the slope that, according to (5), is

(11) $$a_{2}=\frac{1}{\pi^{2}}\sigma_{2}\varphi_{1}\frac{\Gamma_{3}\left(\eta \right)}{\Delta \tau f{\left(h\nu \right)}^{2}w_{0}^{4}}$$

(Supplementary Figures S2–S6). Knowing all of the laser parameters (Methods) and the 2PA cross sections for mCherry [11], we calculate the photochemical quantum yields *φ*_1,m_ from the factor *a*_2_, see Table 1 and Figure 2, bottom.

Interestingly, upon excitation at 1100 nm, the photochemical quantum yield, *φ*_1_ = (1.6 ± 0.3) × 10^−5^, is within experimental error equal to the value previously obtained for mCherry in buffer solution using one-photon excitation (at 532 nm), *φ*_1_ = 1.3 × 10^−5^ [36]. Several important conclusions stem from this result: (1) Our physical model of multiphoton bleaching in thin films catches correctly the main factors. (2) Multiphoton bleaching rate (at least at low intensities) does not depend on local environment of an FP molecule, i.e., buffer vs. *E. coli* cell (cf. [28]). (3) The Kasha’s rule holds at long-wavelength excitation (i.e., the photochemical rate is the same for the excitation accomplished either with one or two photons).

However, in the shorter wavelengths region, 760–1000 nm, the quantum yield *φ*_m_ is ~five to six-fold larger, but does not depend on wavelength. Such violation of Kasha’s rule can be due to lowering of a potential barrier for photochemical reaction when the chromophore gains larger electronic/vibrational energy in the excited state, as discussed in Section 2.1, see Figure 1b. Similar effect was observed for DsRed protein in [37].

With the increasing laser intensity, the dependences in Figure 4 measured at 760, 780, and 800 nm start to grow faster than quadratic, with the power exponent between 3 and 4. When we subtract the *K*^(2)^ = *a*_2_*P*^2^ term (with the *a*_2_ found above) from the observed *K*(*P*) dependence, we find that in a certain power range, where *P* is higher than the threshold, P*, but still is not saturated, the dependence is well described by a 4-th power law: *K*^(4)^ = *K*-*a*_2_*P*^2^ = *a*_4_*P*^4^. The plot of *K*^(4)^ vs. *P*^4^ (Supplementary Figures S7–S9) presents a straight line with the slope *a*_4_ that, according to Equation (9), reads

(12) $$a_{4}=\frac{4}{9\pi^{4}}\sigma_{2}\sigma_{\mathrm{mn}}\sigma_{\mathrm{nc}}\frac{\Gamma_{5}\left(\eta \right)}{\Delta \tau f^{3}{\left(h\nu \right)}^{4}w_{0}^{8}}$$

After substitution of all the laser and geometric parameters, we find a product of two cross sections, *σ*_mn_*σ*_nc_, corresponding to two consecutive steps, see Table 1. This value strongly depends on wavelength, increasing by an order of magnitude upon tuning the laser from 800 to 760 nm.

Once the molecular parameters *σ*_mn_*σ*_nc_ and *φ*_m_ are known, one can predict the P* value if the geometry of the sample illumination and laser parameters (beam waist at the sample, pulse repetition rate, and wavelength) are known. These estimations can provide useful guidelines for researchers aiming to avoid super-quadratic photobleaching. For example, if the multiphoton process at *P* > P* involves four-photon induced reaction, then equating right hand sides of Equations (5) and (9) gives:

(13) $$P^{*}=\frac{3\pi }{2}\sqrt{\frac{\varphi_{m}}{\sigma_{\mathrm{mn}}\sigma_{\mathrm{nc}}}}\sqrt{\frac{\Gamma_{3}}{\Gamma_{5}}}f\;h\nu \;w_{0}^{2}.$$

Using Equation (13), we calculated the predicted threshold power for mCherry for our specific experimental conditions. The results are shown in Table 2 together the with P* values estimated from the “bending” points of the power dependences of Figure 4. The predicted and observed P* values correlate well. This validates our estimation of the laser and sample parameters *w*_0_ and Γ_3_/Γ_5_ entering Equation (13). As one can see, the main factor responsible for the reduction of P* at shorter wavelength is the increase of the *σ*_mn_*σ*_nc_ value.

At even higher laser powers, the *K*(*P*) starts to saturate (i.e., becomes slower than the 4-th power). Fitting the power dependence in this region with the model Function (10) (Supplementary Figure S10) makes it possible to find individual values of the *σ*_mn_ and *σ*_nc_ cross sections, see Table 1.

Knowing these values, we can now compare the bleaching rates of mCherry in two very different experimental settings. (1) A high repetition rate (80 MHz) laser with relatively low pulse energy is focused to a few microns spot onto a thin film of live *E. coli* colony expressing mCherry; and (2) a low repetition rate (1 kHz) regenerative amplifier laser beam with very high pulse energy and uniform power density across the 3 mm diameter passes through a buffer solution of purified mCherry. For the case (2), mCherry bleaches at an initial rate of *k*_1_ = 2.8 × 10^−3^ s^−1^ when the laser intensity is 11.4 W/cm^2^ [28]. It is interesting to check if, by taking the molecular parameters obtained here for bleaching in setting (1), we still would be able to reproduce the rate found in solution with an amplifier setup. The rate *k*_1_ for the homogeneous distribution of power (i.e., without spatial averaging), but with possible saturation (Figure 1d), reads [28]:

(14) $$k_{1}^{\left(4\right)}=\frac{1}{32}\frac{\sigma_{2}\sigma_{\mathrm{nc}}}{\sigma_{\mathrm{mn}}^{3}}\frac{f}{\Delta \tau }\left[\frac{1}{2}X^{3}-X^{2}+X-Xe^{-X}\right],$$

where *X* = *P*/*P*_s_ and *P*_s_ is defined by Equation (8). We find that with a given intensity of an amplifier system (11.4 W/cm^2^) and molecular cross sections taken from Table 1 at 780 nm, saturation parameter is *X* = 4.5, and the resulting *k*_1_ = (2.9 ± 0.9) × 10^−3^ s^−1^, that is virtually the same as measured directly in [28]. This result demonstrates once again that the multiphoton bleaching rate does not depend on local environment of mCherry (*E. coli* cell or buffer). It is also not sensitive to the laser repetition rate even at high laser intensities (i.e., in super-quadratic regime).

### 2.3. Multiphoton Bleaching of mPlum in E. coli Colonies

**Figure 5:**
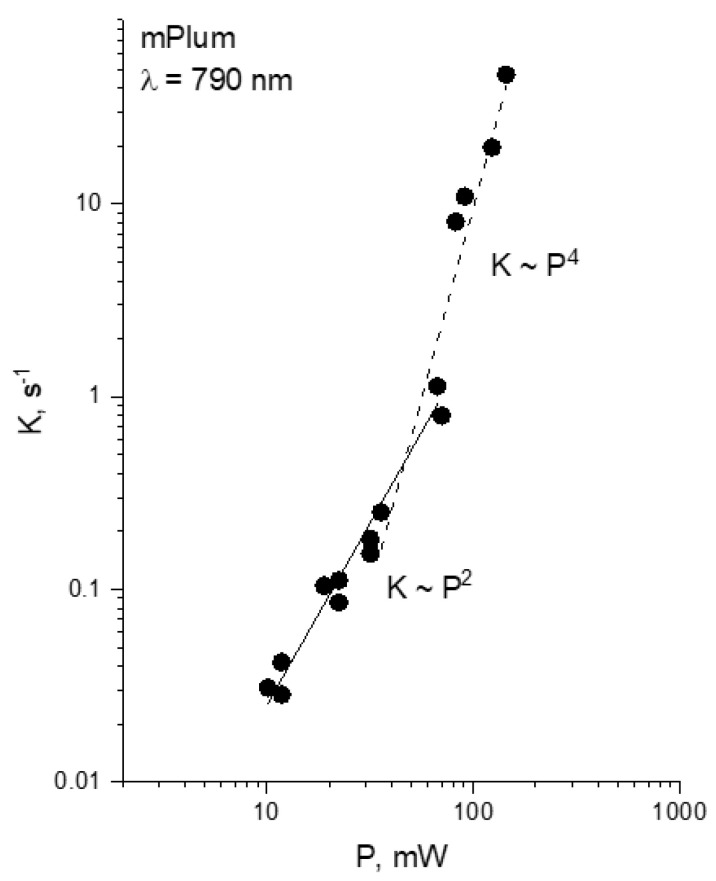
Power dependence of the initial bleaching rate of mPlum at 790 nm, presented in double logarithmic scale.

Figure 5 shows the dependence of photobleaching rate on power for mPlum at 790 nm. Similar to mCherry, it shows a “bending” from the ~*P*^2^ to ~*P*^4^ law occurring at P* = 45 mW (*I*_0_* = 5.2 × 10^28^ photon/cm^2^/s). It has been previously shown that mPlum, similarly to mCherry, bleaches at this wavelength and at *P* = 40–150 mW as a result of four-photon induced electron detachment [28]. The vertical photodetachment energy (5.61 eV) requires photons with λ < 880 nm to initiate the process.

However, the quadratic part has not been observed before [28] because of the used laser powers were above the threshold. We applied the same approach as above to obtain bleaching parameters for both the quadratic and fourth-power regimes (Supplementary Figures S11 and S12). The quantum yield of the two-photon induced reaction is *φ*_m_ = (1.3 ± 0.2) × 10^−4^ (i.e., of the same order as for mCherry in this spectral region). The product of the two consecutive processes cross sections is, *σ*_mn_*σ*_nc_ = (1.6 ± 0.2) × 10^−35^ cm^4^, that is also close to the value obtained for mCherry at 780 nm.

### 2.4. Multiphoton Bleaching of jREX-GECO1 in E. coli Colonies

JREX-GECO1 is a new red genetically encoded Ca^2+^ indicator, described in [35]. In a Ca^2+^-free state, its deprotonated chromophore is weakly fluorescent. Upon binding Ca^2+^, the chromophore becomes protonated and acquires strong fluorescence with a large Stokes shift. It was interesting to see in what state it is present in *E. coli* cells and characterize its multiphoton bleaching properties.

**Figure 6:**
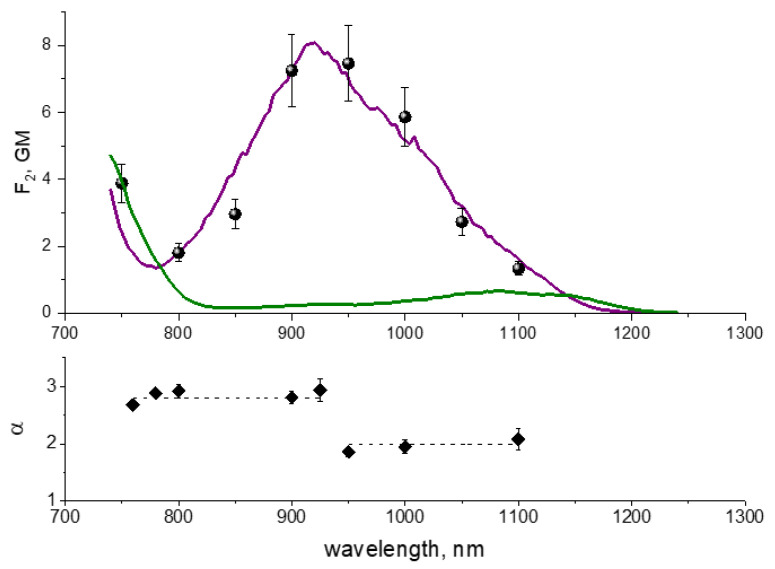
Top: Two photon action spectra of jREX-GECO1 measured in Ca^2+^-free (green line) and Ca^2+^-bound (purple line) states in buffer [35] and in *E. coli* cells (normalized to Ca^2+^-bound spectrum at 900 nm). Bottom: Power exponent of the bleaching rate power dependence as a function of wavelength.

Figure 6 shows that the 2PE spectrum of jREX-GECO1 in *E. coli* cells (circles) closely follows the spectrum of the Ca^2+^-bound state in solution (purple line). This result suggests that in *E. coli* bacteria there is enough Ca^2+^ to saturate the sensor.

The nature of the broad 2PE band peaking at ~920 nm is not completely understood, but it looks similar to that observed in the one-photon absorption spectrum, peaking at ~490 nm [35]. It is assumed that it mainly belongs to a neutral chromophore. However, unusually broad wing at 1000–1100 nm suggests that the whole band could contain two or more hidden electronic transitions [35].

**Figure 7:**
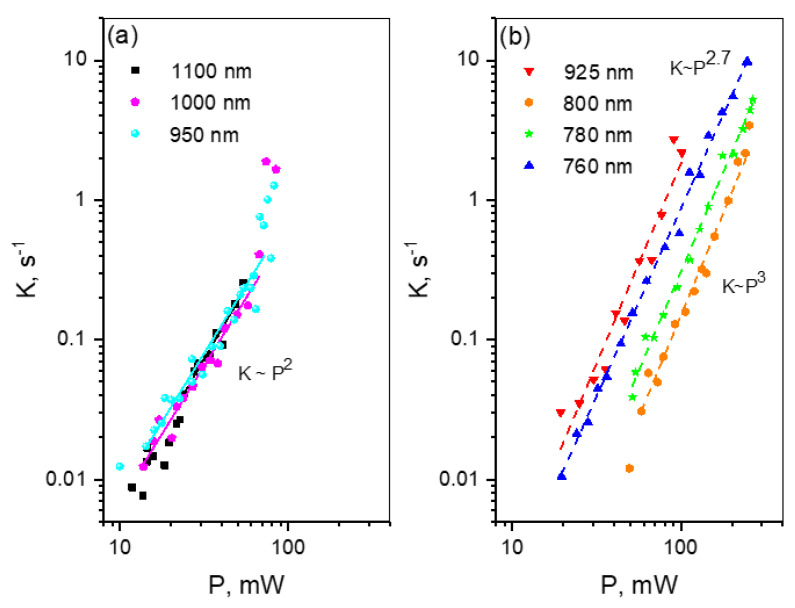
Power dependences of the bleaching rates of jREX-GECO1 at different wavelengths presented in double logarithmic scale. (**a**) Long wavelength range; (**b**) Short wavelength range.

The power dependences of photobleaching rates are very different as compared to mCherry and mPlum. For the wavelengths longer than 925 nm, they are quadratic in the range of ~10–70 mW, see Figure 7a. At *P* > 70 mW, they start growing faster, but we were not able to determine a power exponent for this region because of insufficient range of available powers.

From the quadratic segments of power dependences, we obtained photochemical quantum yields *φ*_1_ by using the same procedure as in Section 2.2 (see Supplementary Figures S13–S15 for the fits). The results show that the quantum yield at 1100 nm is higher than that at 950–1000 nm by a factor of ~4, Table 3. This can be due to a presence of a small fraction of anionic chromophore dominating the absorption at 1100 nm, and bleaching faster than the neutral form (that mainly absorbs at 950–1000 nm).

Upon passing to a shorter wavelength range, a sharp threshold near λ* ≈ 950 nm is observed where the quadratic dependence switches to super-quadratic, see Figure 6, bottom, and Figure 7b. In contrast to mFruits (mCherry and mPlum) where the power exponent is larger than 3 in this spectral region, in jREX-GECO1 it is equal or less than 3. Therefore, we assume that the reaction requires only three photons, not four, and proceeds through a bound electronic state without reaching photodetachment continuum boundary. The wavelength threshold at 950 nm is probably related to a specific resonance where the photon energy becomes sufficient for excitation of this bound state via the S_1_ → S_n_ transition. We believe that the photobleaching mechanism in jREX-GECO1 is different from that in mFruits because the chromophore in the former is neutral (protonated) compared to anionic (deprotonated) one in mFruits. The protonation of chromophore in jREX-GECO1 is facilitated by the substitution of neutral amino acid residue at position 163 (Gln in mCherry or Met in mPlum) with negative Glu (see Supplementary Figure S37 and Table S2) that supposedly comes to a close contact with the chromophore phenolate oxygen in Ca^2+^-bound state. Since the electron detachment energy is much higher for the protonated chromophore than for the deprotonated one, because of strong electrostatic interaction between the electron and remaining molecular frame in the former, the four-photon detachment becomes forbidden there by energy conservation.

The power dependences at 780–925 nm, plotted as *K* vs. *P*^3^, were fitted to Equation (6) (Supplementary Figures S16–S18) to obtain the parameter *σ*_mn_*φ*_n_ from the slope:

(15) $$a_{3}=\frac{4}{5\pi^{3}}\sigma_{2}\sigma_{\mathrm{mn}}\varphi_{n}\frac{\Gamma_{4}\left(\eta \right)}{\Delta \tau f^{2}{\left(h\nu \right)}^{3}w_{0}^{6}}$$

The values of *σ*_mn_*φ*_n_ do not change systematically with the wavelength and fall in the range (0.8–2.0) × 10^−20^ cm^2^. The power dependence at 760 nm is slightly saturated, *K~P*^2.7^ (if the whole range is used for fitting). This makes it possible to obtain parameters *σ*_mn_ and *φ*_n_ separately, see Table 3, by using fitting Function (7), (Supplementary Figure S19). The 760 nm excitation corresponds to a different transition (S_0_ → S_m_), compared to the one at 780–925 nm (S_0_ → S_1_), see Figure 6, and that can probably explain why the value of *σ*_mn_*φ*_n_ is higher at 760 nm than the corresponding average value in the 780–925 region.

### 2.5. Multiphoton Bleaching of tdTomato in E. coli Colonies

**Figure 8:**
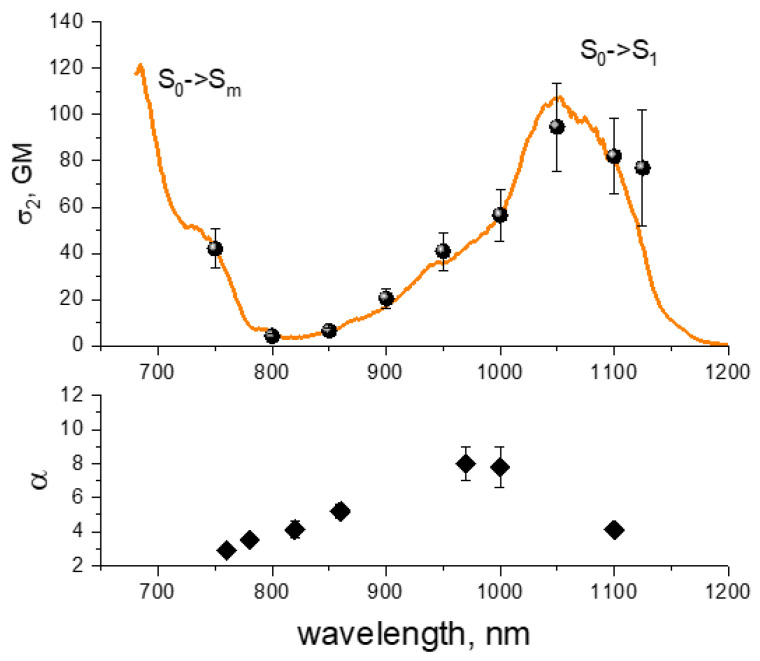
**Top**: Two-photon absorption spectrum of tdTomato in purified buffer solution at pH8, (orange line) [11] and two-photon excitation spectrum of tdTomato measured in *E. coli* cells (symbols) (normalized to the 2PA cross section of solution spectrum at 750 nm). **Bottom**: Power exponent α of the dependence of photobleaching rate on laser power (see text for details).

The 2PE spectral shape of tdTomato in *E. coli* cells overlaps with that previously measured in purified solution at pH8 [11], Figure 8, top, suggesting the expression of this protein in the cells.

**Figure 9:**
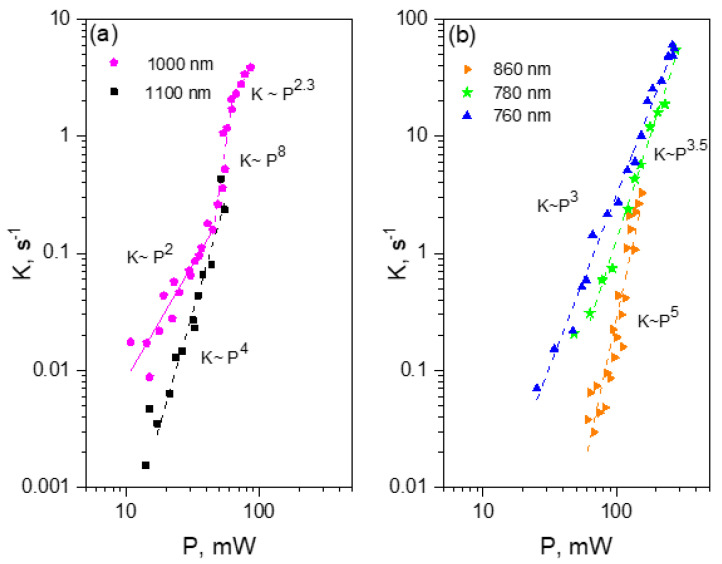
Power dependences of the bleaching rates of tdTomato at different wavelengths (see legends in the insets) presented in double logarithmic scale. (**a**) Long wavelength range; (**b**) Short wavelength range.

Compared to other proteins, tdTomato shows the most diverse and unexpected multiphoton bleaching behavior. When excited at the long-wavelength side of the S_0_ → S_1_ transition at 1100 nm, the power dependence follows a ~*P*^4^ law even at low power levels without any observable *P*^2^ → *P*^4^ transition, Figure 9a, black squares. This behavior is very different from mCherry, where the rate followed the quadratic law at 1100 nm in the same range of powers and the *P*^4^ dependence emerged only for the wavelengths ≤800 nm. This observation suggests that the four-photon electron detachment energy lies much lower in tdTomato than in mCherry. We estimate the VDE < 4*hν* = 4.5 eV (for *hν* = 1.13 eV, corresponding to 1100 nm) for tdTomato vs. VDE = 5.5 eV in mCherry. Physically a decrease in VDE can mean that the electrostatic environment of the chromophore in tdTomato is less electro-positive than in mCherry. The main differences in electrostatic environment of the chromophore in RFPs are due to the charges of residues of amino acids 83, 163, and 215 and their distances from the chromophore [28] (Supplementary Figure S37 and Table S2). In mCherry, residues L83 and E215 are both neutral (E215 is protonated [38] at pH7), and in tdTomato K83 is positive and E215 is negative (deprotonated, according to our alkaline titration experiment, not shown). Although the total charge of the two residues is zero in both proteins, the negative charge of E215 in tdTomato is much closer to the chromophore than the positive charge of K83 (Table S2), which makes the environment of tdTomaato less positive than mCherry. Plotting of *K* vs. *P*^4^ (Supplementary Figure S20) makes it possible to find the value of the product *σ*_mn_*σ*_nc_ at this wavelength, see Table 4.

At shorter wavelengths, 970–1000 nm, the photobleaching rate changes very dramatically as a function of power, see Figure 9a for 1000 nm data (magenta pentagons) and Supplementary Figure S21 for 970 nm data. First, at *P* = 10–40 mW the dependence is quadratic (Supplementary Figure S22), with the quantum yields of the reaction starting from S_1_ state similar to those of mCherry (*φ*_1_ ~10^−5^), see Table 4.

As the laser power exceeds P* = 45 mW (*I*_0_ * = 6.5 × 10^28^ photon/cm^2^/s), the dependence suddenly becomes extremely sharp, following the power law with the exponent *α* = 8 ± 1. To the best of our knowledge, the photobleaching with the involvement of such a high number of photons has not been observed for any organic molecule. Interestingly, the initial (unbleached) fluorescence signal follows a perfect quadratic dependence, Supplementary Figure S23. This suggests that other spurious processes, like harmonic or white continuum generation, can be excluded and most probably the observed kinetics are due to photochemical reaction. When the power reaches ~60 mW, the dependence quickly saturates and becomes either quadratic or slightly super-quadratic, (*α* ≈ 2.3 ± 0.3). This suggests that all the electronic transitions involved in the ladder-type excitation saturate, including the last one that leads to ionization.

An intriguing question of why the four-photon electron detachment observed at longer wavelengths (1100 nm) turns to a much higher-order process at shorter wavelengths remains open and deserves further investigation. We can only speculate that in tdTomato the chromophore can co-exist in the anionic and neutral forms. The anionic form has the 2PE maximum at ~1050–1100 nm and bleaches according to a *K~P*^4^ law. The neutral chromophore, with the 2PE at 900–1000 nm (corresponding to a shoulder at 950 nm in Figure 8, top), bleaches according to a *K*~*P*^8^ law. The RFP chromophore in its protonated (neutral) form has much larger vacuum electron detachment energy compared to deprotonated (anionic) form, VDE = 7.64 vs. 3.27 eV, respectively [39]. The first value translates into the number of photons *n* = VDE/*hν* = 6.2, with *hν* = 1.24 eV for the 1000 nm photon. This suggests that at least seven photons are needed to ionize the RFP chromophore in vacuum, close to our observation. Slightly positive chromophore surrounding in tdTomato, that electrostatically interacts with the positive molecular frame left after ionization, can further increase the VDE, and consequently *n*. We estimate that an electrostatic energy of ~1.2 eV would be enough to make *n* = 8.

At shorter wavelengths, 760–820 nm, the power dependence reflects the *n* = 4 process with moderate (*α* = 3.5 at 780 nm) or strong (*α* = 3.0 at 760 nm) saturation. At these wavelengths, the anionic specie probably dominates in the 2PA spectrum. The corresponding values for the *σ*_mn_ and *σ*_nc_ cross sections are obtained (Supplementary Figures S24–S26) and presented in Table 4. At an intermediate wavelength of 860 nm, we observe the power exponent *α* ≈ 5, which possibly reflects a competition between the bleaching of anionic and neutral chromophore species. The dependence of *α* on wavelength, Figure 8 bottom, shows a resonance at 950–1000 nm, probably depicting the position of neutral chromophore 2PA maximum. It is interesting that a recent paper [40] found that the neutral chromophore of tdTomato has a one-photon absorption peak in the same spectral region, at ~480 nm. However, it is not clear yet to what extent the neutral specie can contribute to the 2PE spectrum.

## 3. Discussion

Our results show that even within a set of red FPs with the same chemical structure of the chromophore, the mechanisms of multiphoton bleaching are quite different. This includes different numbers of photons involved in the process and different power dependences observed at various excitation wavelengths even for a particular protein. Nevertheless, we try to compare here the performance of one protein at different wavelengths or different proteins at similar wavelength using a specific “figure of merit” (FOM) that reflects the maximum possible number of fluorescence photons collected from an FP during a given pixel dwell time in a TPLM experiment. The FOM parameter involves the molecular fluorescence brightness and photobleaching parameters (see Supplementary Information for derivation of equations). In the case of quadratic power dependence of photobleaching rate, the FOM is equal to the ratio of fluorescence quantum yield and photochemical reaction quantum yield starting either from state 1 or m (see Figure 1a,b):

$${\mathrm{FOM}}^{\left(2\right)}=\frac{\varphi_{F}}{\varphi_{1,m}}.$$

For the third-order process without saturation, Figure 1c, the FOM reads

$${\mathrm{FOM}}^{\left(3\right)}=\frac{\varphi_{F}\sigma_{2}^{1/3}}{{\left(\sigma_{\mathrm{mn}}\varphi_{n}\right)}^{2/3}}.$$

In the fourth-order process without saturation, we have

$${\mathrm{FOM}}^{\left(4\right)}=\frac{\varphi_{F}\sigma_{2}^{1/2}}{{\left(\sigma_{\mathrm{mn}}\sigma_{\mathrm{nc}}\right)}^{1/2}}.$$

Using these equations, we estimated the FOM values of the fourth-order process observed experimentally for mCherry, mPlum, and tdTomato at several wavelengths, see Table 5. It should be kept in mind that the comparison between different wavelengths and different proteins using these numbers is possible only for the same laser peak photon flux and the same mechanism of the process.

For the S_0_ → S_m_ transition, excitation of mCherry at 800 nm is slightly beneficial, compared to 780 nm, because of smaller *σ*_mn_*σ*_nc_ parameter for the former. At 790 nm, mPlum shows about two-times smaller FOM ^(4)^ compared to mCherry because of proportionally smaller fluorescence quantum yield. tdTomato has the FOM ^(4)^ similar to mCherry at 780–820 nm, but much larger at 1100 nm. This strong enhancement is due to much higher 2PA cross section of tdTomato compared to mCherry at this wavelength.

Table 6 presents the FOM ^(2)^ values obtained for the RFPs under study in the power ranges where the bleaching rate follows quadratic power dependence.

In this regime, mCherry has much higher FOM ^(2)^ at 1100 nm than at 760–1000 nm. This is explained by much lower *φ*_1_ for the reaction starting from the S_1_ level relatively to S_m_ level. Compared to mCherry, mPlum shows lower FOM ^(2)^ at 790 nm, and jREX-GECO1—higher at 950–1000 nm. Excitation of tdTomato with low powers at 970–1000 nm (Figure 9a), provides extremely large relative FOM ^(2)^ values thanks to high fluorescence quantum yield and relatively low photobleaching yield.

In summary, we developed a method of quantitative characterization of multiphoton bleaching of FPs expressed in *E. coli* colonies that one can use to compare bleaching rates of different mutants in standardized experimental conditions. Our physical model proposed in [28] and further developed here, makes it possible to obtain key molecular parameters involved in photobleaching process. Knowing laser parameters, such as wavelength, pulse duration and peak photon flux in focal plane in addition to molecular bleaching parameters, makes it possible to predict the bleaching rate in a specific experiment. In addition to quantitative estimations of FOM provided above, we can further suggest some general, qualitative, approaches to reduce the bleaching rates. We have shown here, for a first time to the best of our knowledge, that there is often a range of laser photon fluxes where the power dependence of bleaching follows quadratic law. After a critical threshold, *P* = P*, is passed, the dependence turns to a much faster, super-quadratic one that was observed in several previous studies. We suggest that in the TPLM experiments the working laser intensity should be selected close to this threshold to provide the best SBR. We also found that, in agreement with previous observations [20,21,22], it is more advantageous (in terms of brightness/photobleaching ratio) to excite RFPs at their longer wavelength transition, S_0_ → S_1_ (near 1050–1100 nm). In particular, in the case of mCherry, excitation at 1100 nm does not activate a detrimental four-photon electron photodetachment process, and the power dependence follows a quadratic law where any increase in laser intensity provides better SBR ratio, but does not change the brightness/photobleaching rate ratio. At shorter wavelengths, corresponding to a higher, S_0_ → S_m_ transition (760–800 nm), there exists a laser intensity threshold, specific for each wavelength, where the power dependence switches from quadratic to much faster law, corresponding to four-photon process. Such switching was also observed for mPlum.

For the new, red genetically-encoded Ca^2+^-sensor, jREX-GECO1, with a long Stokes shift in the Ca^2+^-bound state, we observe slow photobleaching, following quadratic power law in a broad spectral region from 950–1100 nm. At shorter wavelengths, λ < 950 nm, the bleaching rate starts to grow faster, i.e., according to a third power law. Therefore, excitation at these wavelengths should be avoided.

One of the brightest under two-photon excitation red FP, tdTomato, shows unexpected power dependences of the bleaching rate at some particular wavelengths. At the longest wavelength studied, λ = 1100 nm, the bleaching follows a *K~P*^4^ dependence, reflecting four-photon process. At 970–1000 nm, the dependence starts as quadratic, and then after passing some critical power, becomes very sharp, following the *K~P*^8^ law. At even higher powers, it quickly saturates to become close to quadratic again. At shorter wavelength region, 760–820 nm, the power dependence follows the *K~P*^4^ law with some signs of saturation, typical for mCherry and mPlum. We tentatively explain these observations by a presence of two forms of the chromophore in tdTomato, with the neutral form dominating in the region of 950–1000 nm.

## 4. Materials and Methods

In this work, we studied multiphoton bleaching of RFPs expressed in *E. coli* cells. The cells make colonies on the surface of agar in a square Petri dish. Typically, a colony measures ~1 mm in diameter and ~0.2 mm in thickness. The rod-shaped *E. coli* cell is ~2 μm long with a diameter of <1 μm.

### 4.1. Expressing RFPs in E. coli Colonies

His-tagged RFPs were expressed in DH10B *E. coli* cells with bacterial expression plasmids encoding each protein. mCherry was encoded on the pNCS vector, which contains a promoter for constitutive expression. tdTomato, mPlum, and jREX-GECO1 were encoded on pBAD. tdTomato-pBAD was a gift from Michael Davidson and Nathan Shaner and Roger Tsien (Addgene plasmid # 54856; http://n2t.net/addgene:54856 (accessed on 23 December 2021); RRID:Addgene_54856), mPlum-pBAD was a gift from Michael Davidson and Roger Tsien (Addgene plasmid # 54564; http://n2t.net/addgene:54564 (accessed on 23 December 2021); RRID:Addgene_54564). jREX-GECO1 was grown into a modified pBAD backbone pTorPE with restriction cloning. jpTorPE-jREX-GECO1 was a gift from Robert Campbell and Thomas Hughes (Addgene plasmid # 113941; http://n2t.net/addgene:113941 (accessed on 23 December 2021); RRID:Addgene_113941). *E. coli* expressing an RFP were cultured in Terrific Broth (BD, Sparks, MD, USA) for 18 h at 37°. To induce expression of the pBAD plasmids, 0.1% *w*/*v* of L-arabinose was added to the media before inoculating.

### 4.2. Preparing Plates with E. coli Colonies

Square 10 × 10 cm Petri dishes (Simport, Beloeil, QC, Canada) were filled with 80 mL of LB agar at 50 °C with the appropriate antibiotic and, in the case of pBAD vector expression, 0.02% *w*/*v* of L-arabinose added. The plates were set on a horizontal surface for 20 min. Transformed *E. coli* suspension was diluted 1:10, 1:100, and 1:1000 times with Terrific Broth and were spread onto the plate using 3 mm glass beads (Fisher Scientific, Walthman, MA, USA), and then the plates were incubated for 20 h at 37 °C. After incubation, the plates with colonies were put in refrigerator at 4 °C for 2 days. After 2 days, the colonies acquired a color and fluorescence corresponding to a mature RFP chromophore and were ready for optical experiments. The plates with *E. coli* colonies could be stored at 4 °C for a few months without losing the ability to fluoresce.

### 4.3. Optical Instrument

The customized optical instrument, called the GIZMO, was previously designed and implemented for screening of large libraries of FP mutants for their two-photon brightness. It was described in detail in [41]. Its complete CAD model as well as software and analysis programs can be found at github.com/rosanamolina/gizmo-paper (accessed on 23 December 2021). Here we used this instrument to characterize multiphoton bleaching parameters of a particular FP expressed in several colonies on the same plate. Briefly, the GIZMO consists of two optical arms used for one- and two-photon excitation, respectively. The micrometer stage can move the sample (a Petri dish) between the two arms. While in the one-photon arm, a brief illumination of the dish with an LED array provides fluorescence of individual colonies, captured with a CCD camera. The *x* and *y* coordinates of each colony are then accurately represented in the image. The two-photon arm is a variant of two-photon laser scanning microscope. A femtosecond laser (Insight DeepSee, Spectra-Physics, Milpitas, CA, USA) beam first gets attenuated with a computer-controlled Pockels cell, then passing through two galvo mirrors, a scan lens, and a tube lens, hits the objective lens mounted on a piezo stage allowing to scan the focal spot of a laser in *z*-direction with a range of 400 μm. The image obtained at the one-photon arm is used to bring a particular colony into the laser focus, using the *x*–*y* movement of the stage. The fluorescence is collected by the same objective and directed through a reflecting dichroic mirror and a combination of a 570–680 nm band pass and a 745 nm short pass filters to PMT.

### 4.4. Photobleaching Experiments

The customized photobleaching program (written in MATLAB) makes it possible to run photobleaching kinetics consecutively at six different spots (*x*,*y*,*z*) inside one colony. We first selected a bright colony on the plate by using an image acquired in the one-photon arm, then in the two-photon arm moved the stage in the *x* and *y* directions, such that the center of this colony came to the laser beam axis. Next, we adjusted the *z* position of the stage to get the strongest fluorescence signal. The photobleaching program calculated the coordinates (*x*,*y*) of six points arranged in the apices of a hexagon with the diameter of 80 μm around the initial point. At each point, the program found an optimum *z* position (with maximum fluorescence signal) and then at this position recorded the fluorescence decay curve with a particular, program-controlled (with Pockels cell) laser power, and then moved sequentially to the next spots where it recorded the decay at different power levels. In most of the cases we used 10 s for a dwell time per point, but for some slow kinetics, 30 s. Typically, the binning of 100 data points (with averaging) was used to get totally 25,000 points per 10 s of scan. The program runs on top of a legacy version of ScanImage (Vidrio Technologies, Ashburn, VA, USA). In these bleaching experiments we used a low numerical aperture objective lens, Plan-Neofluar 2.5 ×/0.075 (Carl Zeiss, White Plains, NY, USA). A low NA value helps to keep the laser waist diameter in focus (7–11 μm), much larger than the size of an *E. coli* cell (0.5–2 μm). Homogeneous irradiation of a cell allows disregarding of diffusion artifacts in quantitative description of bleaching kinetics. This particular lens has a good transmittance in NIR and its low NA causes less group velocity dispersion (GVD) [42].

### 4.5. Characterizing Laser Beam Parameters in Focal Area

The laser waist *w* in focal area was measured with the razor blade method. The razor was fixed onto a GIZMO micron stage and the power meter was mounted underneath. The stage was moved in *x* direction at a fixed *z* position. The resulting dependence of power on *x* was fit to a function

$$P\left(x\right)=A\int_{-\infty }^{x}\exp\left(-\frac{2{\left(t-x_{c}\right)}^{2}}{w^{2}}\right)dt.$$

This scan was performed at several positions of *z*, and the resulting dependence of *w* on *z* was plotted and fit to a function

$$w\left(z\right)=w_{0}\sqrt{1+{\left(\frac{\lambda \left(z-z_{0}\right)}{\pi w_{0}^{2}}\right)}^{2}}$$

The best-fit parameters *z*_0_ and *w*_0_ provide the focal plane position and the beam waist radius at focal plane, respectively. This procedure was repeated for several laser wavelengths, and the dependence of *w*_0_ on wavelength is shown in Supplementary Figure S27. The resulting waist parameter *w*_0_ varies between 3.5 and 5.5 μm. The Rayleigh length, $z_{R}=\pi w_{0}^{2}/\lambda$, was calculated and plotted as a function of *λ*, see Supplementary Figure S28. The focal spot size (*w*_0_) is a critical parameter in calculating multiphoton bleaching cross sections and quantum yields. The razor blade method used here could give a distorted *w*_0_ value because of diffraction effects if the beam size on the back aperture of objective lens is not very small [43]. Therefore, we developed and employed an independent method of direct evaluation of *z*_R_ and *w*_0_ in *E. coli* colonies, Section 4.6.

The pulse duration at 1100 nm was estimated as the transform-limited value for the Gaussian pulse shape. Using spectral widths measurements (Ocean Optics spectrometer, Ocean Insight, Rochester, NY, USA) of the laser output from 850 to 1000 nm, we obtained an almost constant value of pulse duration, ∆τ = (109 ± 1) fs. We assume that the pulse duration after the beam expander and objective lens in our optical system does not acquire any GVD broadening at 1100 nm [42] and, therefore, is equal to 109 fs. To evaluate the relative pulse duration ∆*τ* as a function of wavelength at the sample positions, we measured the two-photon excited fluorescence signal from a solution of Rhodamine 6G in methanol, cf. [23]. The solution, contained in a 1 mm thick optical cuvette, was placed in the focus of the objective lens and the power *P* was kept constant when going from one wavelength to another. In this situation, the thickness of layer *l* was much larger than the Rayleigh length, *l* >> *z*_R_, and integrated fluorescence signal over time and space (taking into account the Gaussian-Lorentzian intensity distribution (2)), reads

$$F_{2}=C\sigma_{2}\left(\lambda \right)\frac{\lambda }{\Delta \tau \left(\lambda \right)}P^{2}\;,$$

where *C* is a constant. To find the pulse duration as a function of wavelength, we regroup the previous equation to obtain:

$$\Delta \tau \left(\lambda \right)=CP^{2}\lambda \frac{\sigma_{2}\left(\lambda \right)}{F_{2}\left(\lambda \right)}.$$

Figure S29 shows the ∆τ values calculated according to this equation (with the *σ*_2_(*λ*) values taken from [44]), and normalized to the value of 109 fs at 1100 nm.

### 4.6. Colony Thickness, Rayleigh Length, and Focal Spot Size inside a Colony

To measure colony thickness and independently estimate the Rayleigh length, we performed a *z*-scan of fluorescence signal across several colonies expressing different FPs. This was done by changing the *z* position with a step of 200 or 250 μm (using the stage movement) and scanning a 400 μm region with the piezo scan of the lens. Those 400 μm segments were then “glued” together to obtain the *z*-profile of fluorescence intensity, Supplementary Figures S30–S35. These profiles were normalized to 1 and fitted to a model function that is obtained as a convolution of the Gaussian-Lorentzian distribution squared and a rectangular function of width *l*, describing the distribution of fluorophores inside a colony, see Supplementary Data. Colony thicknesses vary between 150 and 240 μm. The Rayleigh range obtained inside the colonies is ~30% larger than that in air, found in Section 5. We explain this effect by the dependence of wavelength of light on refractive index of medium. In fact, the general expression for *z*_R_ in a medium reads [45]:

$$z_{R}=\frac{n_{0}\pi w_{0}^{2}}{\lambda }$$

where *n*_0_ is the refractive index of medium and λ is the wavelength of light in vacuum. Since the refractive index of water is 1.33, the *z*_R_ measured in a colony should be 1.33 times larger than in air, as observed (Supplementary Figure S28). Finally, we calculate the *w*_0_ values from the *z*_R_ measured in colonies, as

$$w_{0}=\sqrt{\lambda z_{R}/n_{0}\pi }.$$

The results match quite well those obtained with the razor blade method, see Figure S27.

### 4.7. Two-Photon Excitation Spectra of RFPs inside E. coli Colonies

To obtain relative shape of the 2PE spectrum of an RFPP inside a colony, we first measured its two-photon excited fluorescence signal *F*_2,S_(λ) (100 ms acquisition time, no bleaching) at several excitation wavelengths. In this measurement, position *z* was adjusted to give the maximum signal (different at different λ). Then, exactly in the same excitation conditions (laser power) we measured the two-photon excited signal from the solution of Rhodamine 6G in methanol in 1 mm cuvette, *F*_2,R_(λ). The cuvette was mounted inside an empty Petri dish and placed in focus of the laser instead of the plate with colonies. The *z* position was again adjusted to the maximum signal. This sample was used as a reference to calibrate variations of the laser power and beam spatial and temporal profile as a function of wavelength. The corrected 2PE spectrum of an RFP sample in cells was then calculated according to

$$f_{2PE,S}\left(\lambda \right)=\frac{F_{2,S}\left(\lambda \right)}{F_{2,R}\left(\lambda \right)}f_{2PE,R}\left(\lambda \right),$$

where $f_{2PE,R}\left(\lambda \right)$ is the corrected 2PE spectrum of the reference solution, presented in [44].

## Figures and Tables

**Table 1:** Molecular photobleaching parameters of mCherry at different wavelengths.

λ, nm	n	*σ*_2_, GM ^(a)^	*φ*_1,m_ ^(b)^	*σ*_mn_*σ*_nc_, cm^4 (c)^	*σ*_mn_, cm^2 (d)^	*σ*_nc_, cm^2 (d)^
1100	2	24	(1.6 ± 0.3) × 10^−5^	--	--	--
1000	2	6.2	(1.0 ± 0.2) × 10^−4^	--	--	--
800	2 to 4	22	(1.0 ± 0.2) × 10^−4^	(5.0 ± 0.8) × 10^−36^	(3.7 ± 1.4) × 10^−17^	(1.3 ± 0.5) × 10^−19^ *
780	2 to 4	42	(8.5 ± 1.7) × 10^−5^	(1.7 ± 0.3) × 10^−35^	(5.0 ± 1.6) × 10^−17^	(3.4 ± 0.9) × 10^−19^
760	2 to 4	71	(1.0 ± 0.2) × 10^−4^	(3.3 ± 0.5) × 10^−35^	(8.8 ± 1.9) × 10^−17^	(7.3 ± 1.2) × 10^−19^

Column 2 shows the number of photons *n* involved in the process. ^(a)^ 2PA cross sections are taken from [11]. ^(b)^ Found from the fits of *K*(*P*) dependences to Function (5) in the low power range below the threshold. ^(c)^ Found from the fits of *K*(*P*) dependences to Function (9) in the power range just above the threshold. ^(d)^ Found from the fits of *K*(*P*) dependences to Function (10) in the power range above the threshold. * Obtained by dividing *σ*_mn_*σ*_nc_ (column 5) by *σ*_mn_ (column 6).

**Table 2:** Threshold laser powers P* and peak photon flux *I*_0_* for the transition from quadratic to super-quadratic dependence of the photobleaching rate for mCherry.

λ, nm	(*φ*_m_/*σ*_mn_*σ*_nc_)^1/2^, cm^−2^	*w*_0_^2^, cm^2^	P* Predicted, mW (*I*_0_*, photons/cm^2^/s)	P* Observed, mW (*I*_0_*, photons/cm^2^/s)
760	1.7 × 10^15^	1.32 × 10^−7^	26 (3.6 × 10^28^)	19 (2.6 × 10^28^)
780	2.2 × 10^15^	1.37 × 10^−7^	34 (3.9 × 10^28^)	28 (3.2 × 10^28^)
800	4.5 × 10^15^	1.43 × 10^−7^	68 (5.4 × 10^28^)	61 (4.8 × 10^28^)

Column 2 presents a combination of molecular parameters that define the P* value. Column 3 shows the laser waist squared that is also responsible for P*.

**Table 3:** Molecular photobleaching parameters of jREX-GECO1 at different wavelengths.

λ, nm	*n*	*σ*_2_, GM ^(a)^	*φ*_1_ ^(b)^	*σ*_mn_*φ*_n_, cm^2 (c)^	*σ*_mn_, cm^2 (d)^	*φ*_n_
1100	2	7.9	(2.1 ± 0.3) × 10^−4^	--	--	--
1000	2	25	(5.7 ± 0.9) × 10^−5^	--	--	--
950	2	34	(5.0 ± 0.8) × 10^−5^	--	--	--
925	3	38	--	(1.2 ± 0.2) × 10^−20^ (m = 1)	--	--
900	3	35		(2.0 ± 0.3) × 10^−20^ (m = 1)	--	--
800	3	8.8	--	(7.7 ± 1.1) × 10^−21^ (m = 1)	--	--
780	3	6.5	--	(1.5 ± 0.2) × 10^−20^ (m = 1)	--	--
760	3	8.7	--	(3.0 ± 0.5) × 10^−20^ (m > 1)	(1.4 ± 0.4) × 10^−17^	(2.2 ± 0.7) × 10^−3^

Column 2 shows the number of photons *n* involved in the process. ^(a)^ 2PA cross sections of Ca^2+^-saturated state are taken from [35]. ^(b)^ Found from the fits of *K*(*P*) dependences to Function (5) in power range below the threshold. ^(c)^ Found from the fits of *K*(*P*) dependences to Function (6). ^(d)^ Found from the fits of *K*(*P*) dependences to Function (7).

**Table 4:** Molecular photobleaching parameters of tdTomato at different wavelengths.

λ, nm	n	*σ*_2_, GM ^(a)^	*φ*_1,m_ ^(b)^	*σ*_mn_*σ*_nc_, cm^4 (c)^	*σ*_mn_, cm^2 (d)^	*σ*_nc_, cm^2 (d)^
1100	4	80	--	(2.3 ± 0.4) × 10^−36^	--	--
1000	2 to 8	56	(3.7 ± 0.6) × 10^−5^	--	--	--
970	2 to 10	42	(1.6 ± 0.2) × 10^−5^	--		
860	5	22	--	--	--	--
820	4	3.2	--	(4.3 ± 0.2) × 10^−36^	--	--
780	4	8.7	--	(1.6 ± 0.8) × 10^−35^	(4.3 ± 1.5) × 10^−17^	(3.8 ± 1.3) × 10^−20^
760	4 (α = 3)	31	--	--	--	(4.8 ± 0.7) × 10^−19^

Column 2 shows the number of photons *n* involved in the process. ^(a)^ 2PA cross sections are taken from [11]. ^(b)^ Found from the fits of *K*(*P*) dependences to Function (5) in power range below the threshold. ^(c)^ Found from the fits of *K*(*P*) dependences to Function (9). ^(d)^ Found from the fits of *K*(*P*) dependences to Function (10).

**Table 5:** Figure of Merit for TPLM applications of FPs showing fourth-order power dependence of multiphoton bleaching rate.

Protein	λ, nm	*σ*_2_, GM ^(a)^	*σ*_mn_*σ*_nc_, cm^4^	*φ*_F_ ^(b)^	FOM ^(4)^ [×10^−8^]
mCherry	800	22	5.0 × 10^−36^	0.24	5
	780	42	1.7 × 10^−35^	0.24	3.8
	760	71	3.3 × 10^−35^	0.24	3.5
mPlum	790	43	1.6 × 10^−35^	0.13	2.1
tdTomato	1100	80	2.3 × 10^−36^	0.72	43
	820	3.2	4.3 × 10^−36^	0.72	6.2
	780	8.7	1.6 × 10^−35^	0.72	5.3

^(a)^ Taken from [11]. ^(b)^ Taken from [10].

**Table 6:** Figure of Merit for TPLM applications of FPs showing second-order power dependence of multiphoton bleaching rate.

Protein	λ, nm	*φ*_1,m_^(a)^	*φ*_F_^(b)^	FOM ^(2)^ [×10^3^]
mCherry	1100	1.6 × 10^−5^	0.24	15
	1000	1.0 × 10^−4^	0.24	2.4
	800	1.0 × 10^−4^	0.24	2.4
	780	8.5 × 10^−5^	0.24	2.8
	760	1.0 × 10^−4^	0.24	2.4
mPlum	790	1.3 × 10^−4^	0.13	1
jREX-GECO1	1100	2.1 × 10^−4^	0.21	1
tdTomato	1000	5.7 × 10^−5^	0.21	3.7
	950	5.0 × 10^−5^	0.21	4.2
	1000	3.7 × 10^−5^	0.72	20
	970	1.6 × 10^−5^	0.72	45

^(a)^ Taken from [11]. ^(b)^ For mFruits and tdTomato taken from [10] and for jREX-GECO1—from [35].

## Supplementary Materials

The following supporting information can be downloaded at: https://www.mdpi.com/article/10.3390/ijms23020770/s1.
