# Supplementary material for: Multiphoton Bleaching of Red Fluorescent Proteins and the Ways to Reduce It
Source: Int J Mol Sci. 2022 Jan 11;23(2):770. doi: 10.3390/ijms23020770 (PMC8775990; doi:10.3390/ijms23020770)
Supplement: Supplementary file 1 [file ijms-23-00770-s001.zip › ijms-1547362-supplementary.pdf]

Supplementary Information

1. *Functions $\Gamma_i(\eta)$ entering the expressions for photobleaching rates of different order.*

These functions reflect the geometry of illumination and depend on the dimensionless parameter equal to the ratio of colony half-thickness $l/2$ and Rayleigh length of Gaussian beam inside a colony, z_R. If we define $\eta = l/2z_R$, we obtain (see definition of functions $\gamma(l,z_R)$ in [Drobizhev, 2014]):

$$\Gamma_2(\eta) = \frac{\gamma_2(l,z_R)}{\gamma_1(l,z_R)} = \frac{1}{2}\left(\frac{\eta}{(1+\eta^2)\arctan(\eta)} + 1\right)$$

$$\Gamma_3(\eta) = \frac{\gamma_3(l,z_R)}{\gamma_1(l,z_R)} = \frac{\eta}{4(1+\eta^2)^2\arctan(\eta)} + \frac{3}{8}\left(\frac{\eta}{(1+\eta^2)\arctan(\eta)} + 1\right)$$

$$\Gamma_4(\eta) = \frac{\gamma_4(l,z_R)}{\gamma_1(l,z_R)} = \frac{1}{\arctan(\eta)}\left(\frac{\eta}{6(1+\eta^2)^3} + \frac{5\eta}{24(1+\eta^2)^2}\right) + \frac{5}{16}\left(\frac{\eta}{(1+\eta^2)\arctan(\eta)} + 1\right)$$

$$\Gamma_5(\eta) = \frac{\gamma_5(l,z_R)}{\gamma_1(l,z_R)} = \frac{1}{\arctan(\eta)}\left(\frac{\eta}{8(1+\eta^2)^4} + \frac{7\eta}{48(1+\eta^2)^3} + \frac{35\eta}{192(1+\eta^2)^2}\right) + \frac{35}{128}\left(\frac{\eta}{(1+\eta^2)\arctan(\eta)} + 1\right)$$

All the $\Gamma(\eta)$ functions and the ratio $\Gamma_3(\eta)/\Gamma_5(\eta)$ are presented in Figure S1.

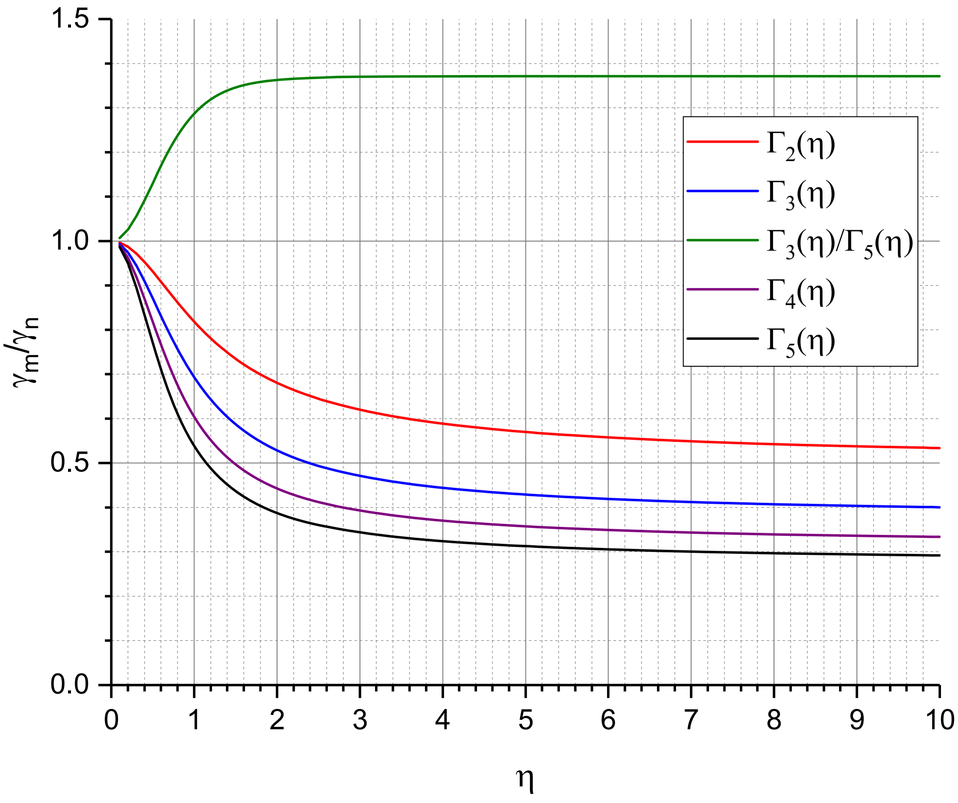

Figure S1. All $\Gamma_i(\eta)$ functions involved in the macroscopic photobleaching rates of different order.

2. The dependences of bleaching rate K on P^2 for mCherry at different wavelengths

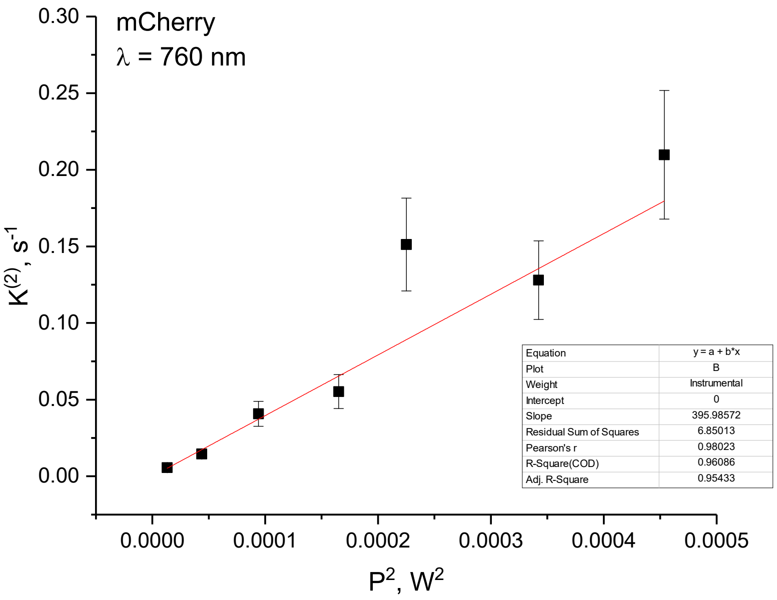

Figure S2. Bleaching rate of mCherry at 760 nm in the low power range, presented as a function of P^2. Linear fit is shown by the red line.

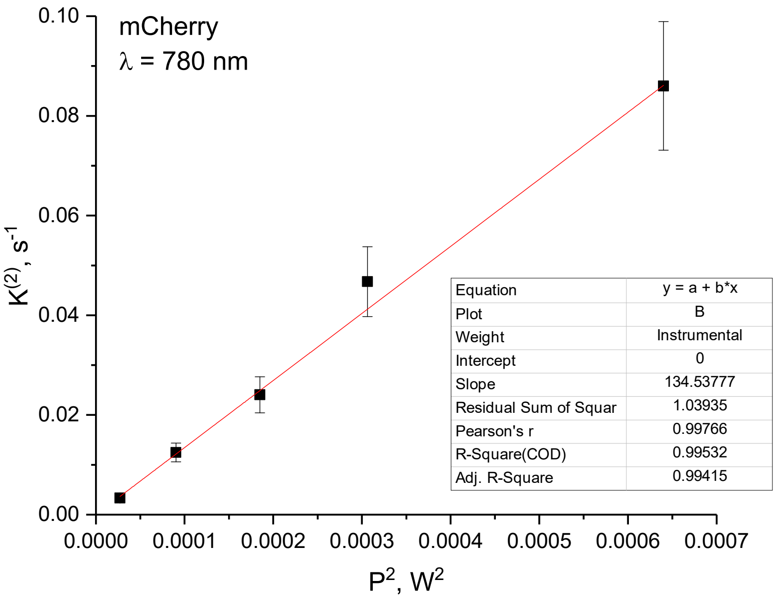

Figure S3. Same as in Fig. S2, but for 780 nm.

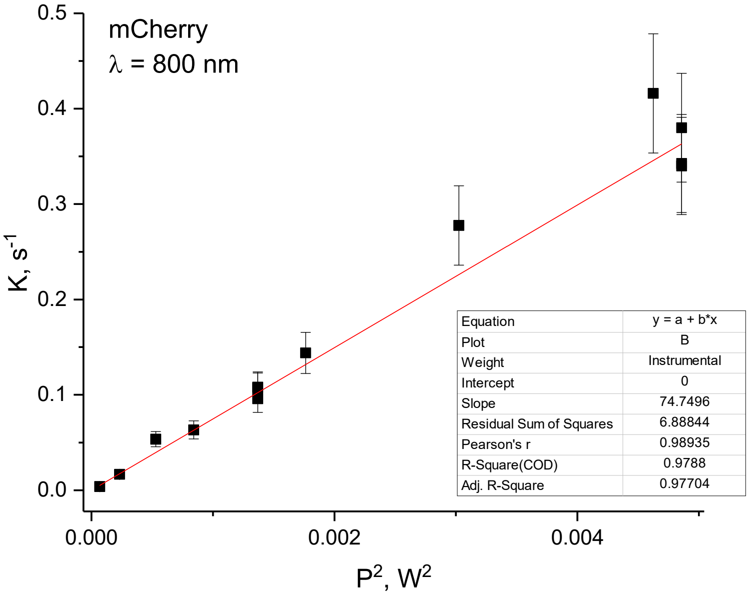

Figure S4. Same as in Fig. S2, but for 800 nm.

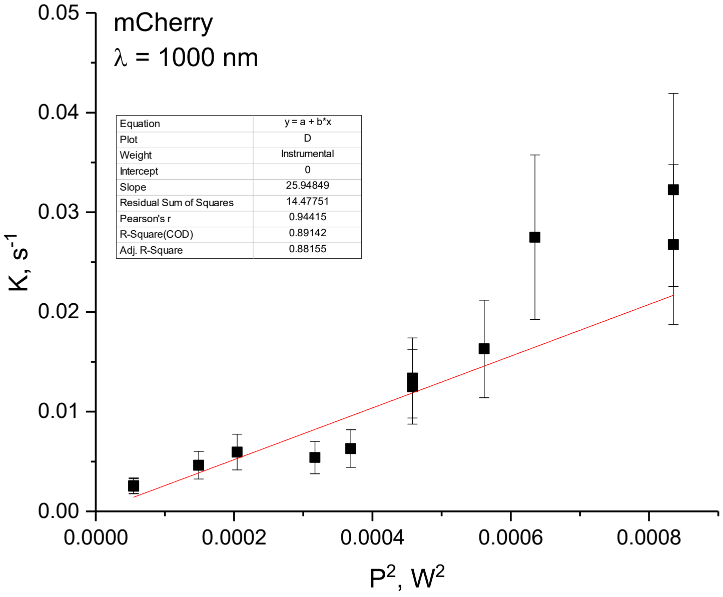

Figure S5. Same as in Fig. S2, but for 1000 nm.

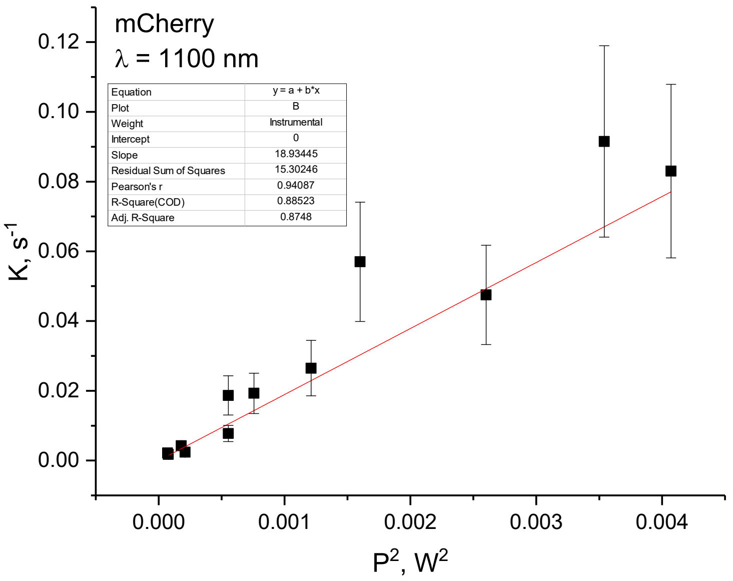

Figure S6. Same as in Fig. S2, but for 1100 nm.

3. *The dependences of bleaching rate K on P^4 for mCherry at different wavelengths*

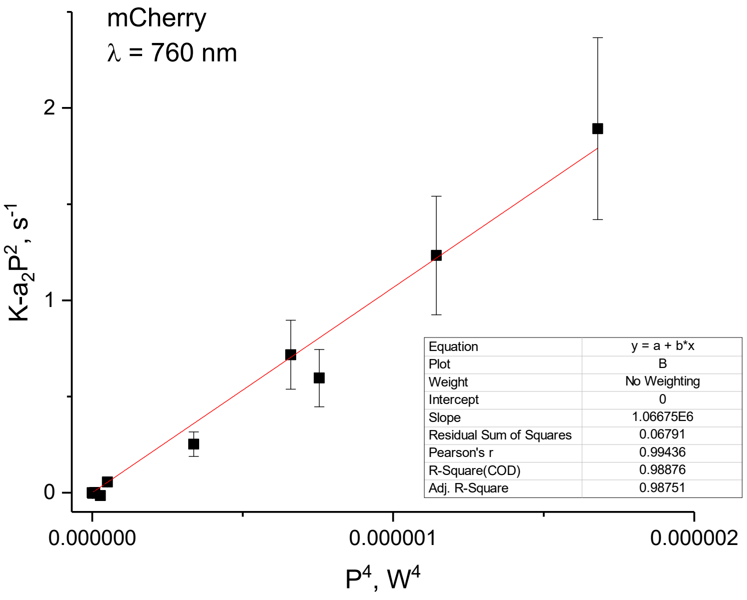

Figure S7. Bleaching rate with subtracted quadratic contribution (see section 2 above), $K - a_2P^2$, versus P^4 for mCherry at 760 nm in the region of powers higher than the P* threshold, but lower than where the $K \sim P^4$ dependence starts to saturate. The linear fit is shown by the red line.

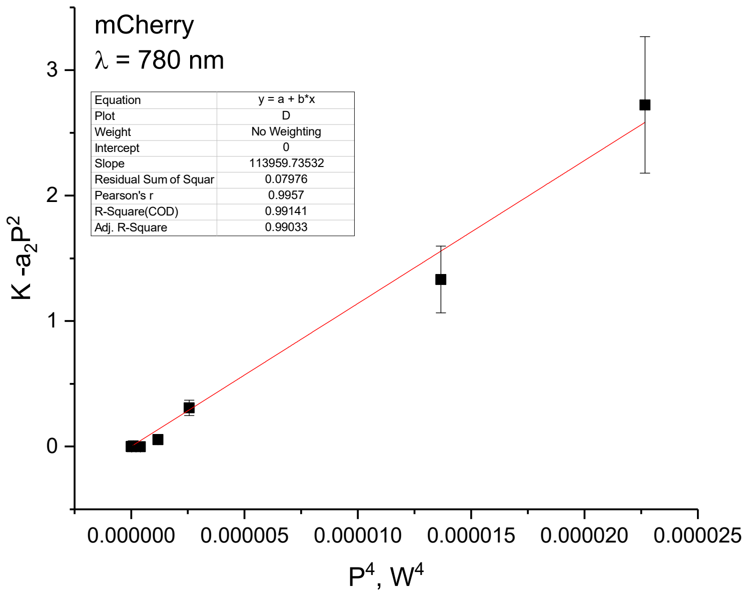

Figure S8. Same as in Fig. S7, but for λ = 780 nm.

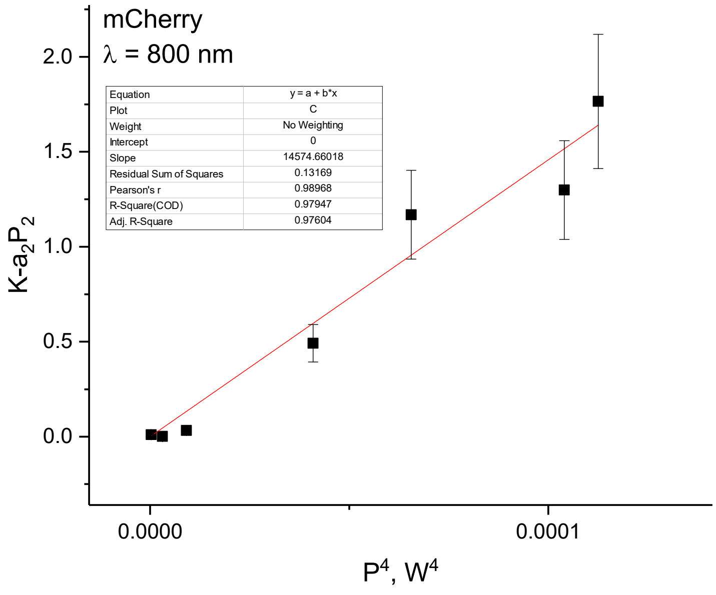

Figure S9. Same as in Fig. S7, but for λ = 800 nm.

4. The dependence of bleaching rate K on P for mCherry at 760 nm in the saturation regime

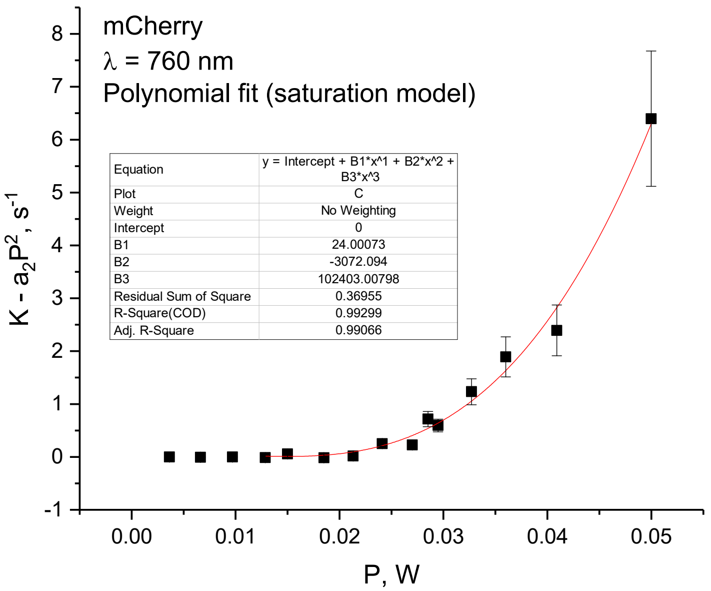

Figure S10. Bleaching rate with subtracted quadratic contribution (see section 2 above), $K - a_2P^2$, versus P for mCherry at 760 nm in the region of powers where the $K \sim P^4$ dependence starts to saturate. The fit to a third order polynomial, according to eq. (10), is shown by the red line.

5. *The dependence of bleaching rate K on* P^2 *for mPlum at 790 nm*

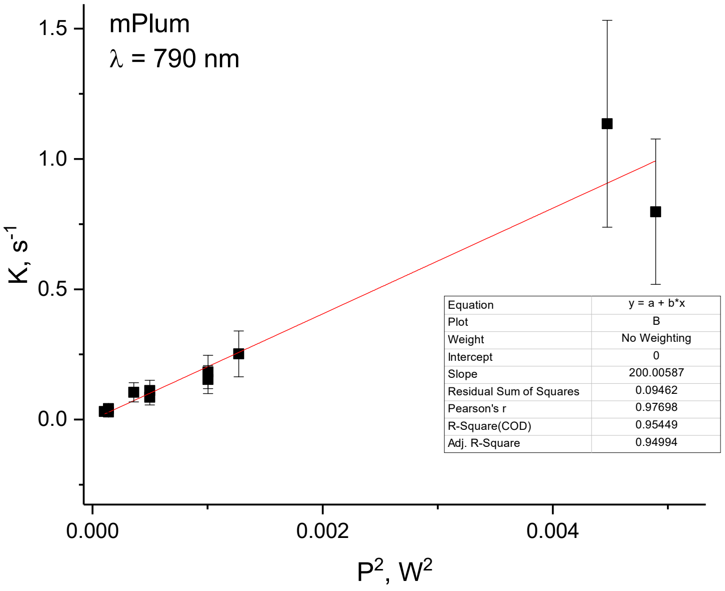

Figure S11. Bleaching rate of mPlum at 790 nm in the low power range, presented as a function of P^2. Linear fit is shown by a red line.

6. *The dependence of bleaching rate K on P^4 for mPlum at 790 nm*

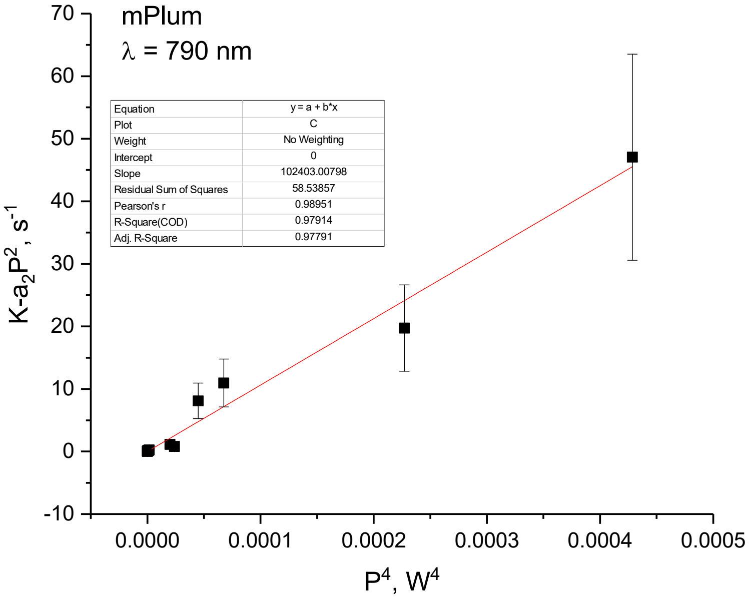

Figure S12. Bleaching rate with subtracted quadratic contribution (see section 5 above), $K - a_2P^2$, versus P^4 for mPlum at 790 nm in the region of powers higher than the P* threshold, but lower than where the $K \sim P^4$ dependence starts to saturate. The linear fit is shown by the red line.

7. *The dependences of bleaching rate K on P^2 for jREX-GECO1 at different wavelengths*

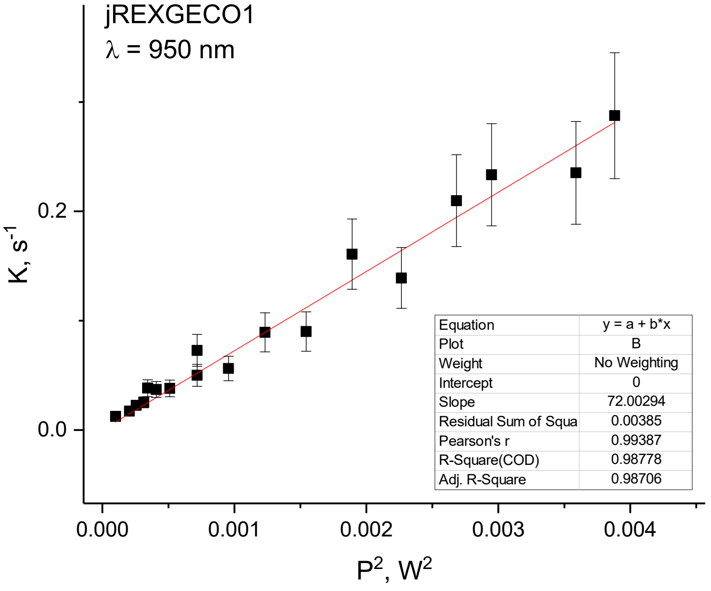

Fig. S13. Bleaching rate of jREX-GECO1 at 950 nm in the low power range, presented as a function of P^2. Linear fit is shown by the red line.

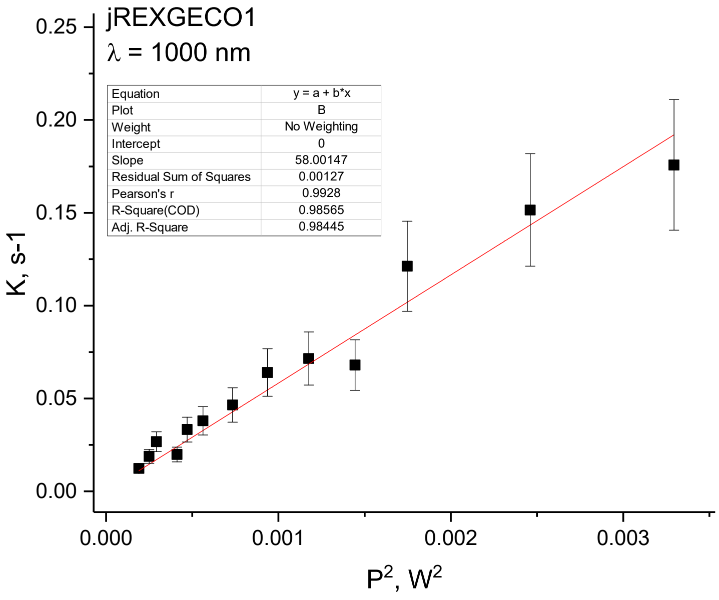

Figure S14. Same as in Fig. S13, but for 1000 nm.

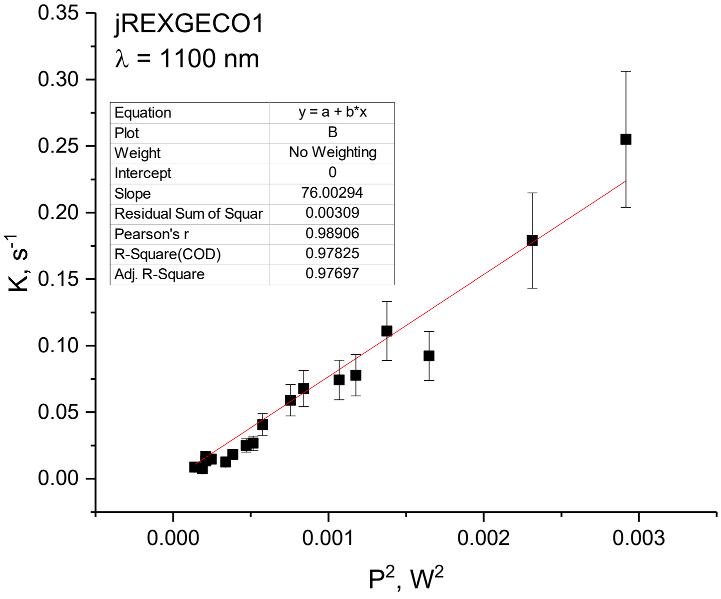

Figure S15. Same as in Fig. S13, but for 1100 nm.

8. *The dependences of bleaching rate K on P^3 for jREX-GECO1 at different wavelengths*

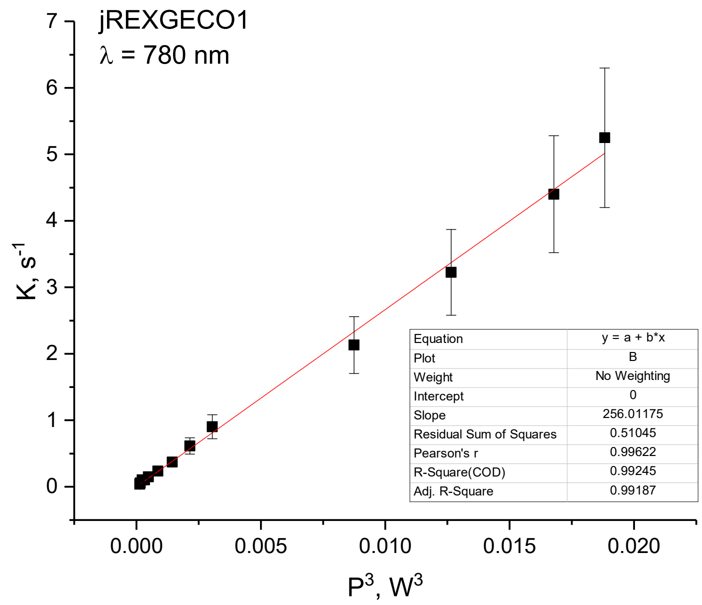

Figure S16. Bleaching rate versus P^3 for jREX-GECO1 at 780. The linear fit is shown by the red line.

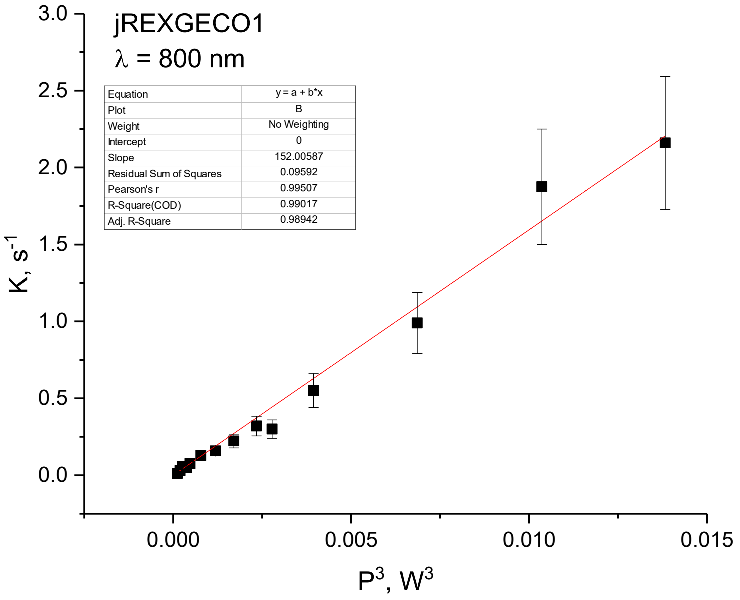

Figure S17. Same as in Fig. S16, but for 800 nm.

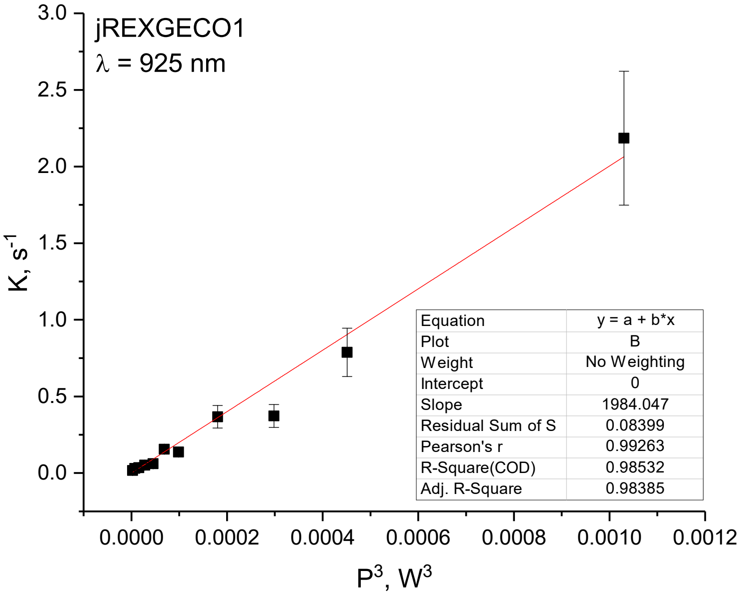

Figure S18. Same as in Fig. S16, but for 925 nm.

9. *The dependence of bleaching rate K on P for jREX-GECO1 at 760 nm in the saturation regime*

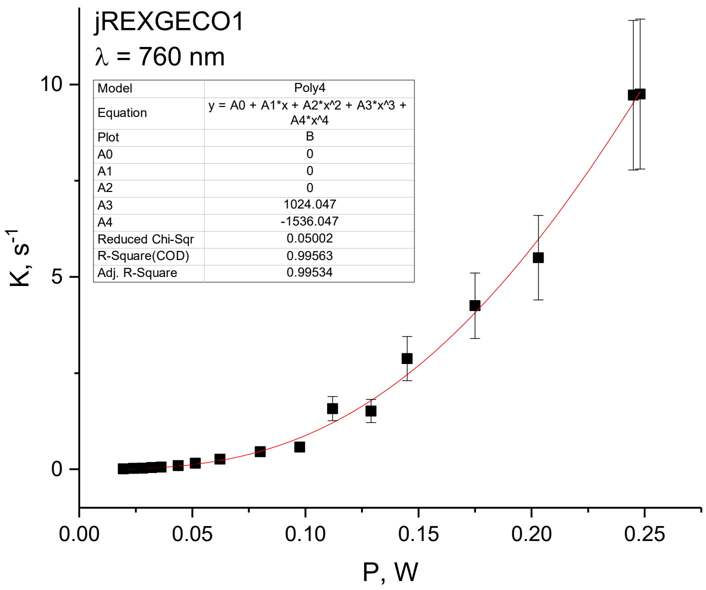

Figure S19. Bleaching rate versus *P* for jREX-GECO1 at 760 nm in the region of powers where the *K* ~ P^3 dependence starts to saturate. The fit to a fourth order polynomial, according to eq. (7), is shown by the red line.

10. *The dependence of bleaching rate K on P^4 for tdTomato at 1100 nm*

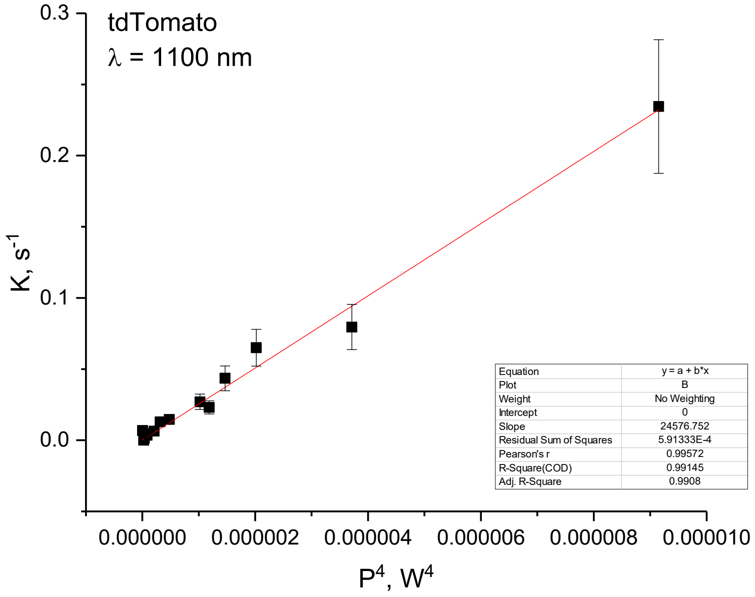

Figure S20. Bleaching rate K versus P^4 for tdTomato at 1100 nm. The linear fit is shown by the red line.

11. The dependence of bleaching rate K on P for tdTomato at 970 nm

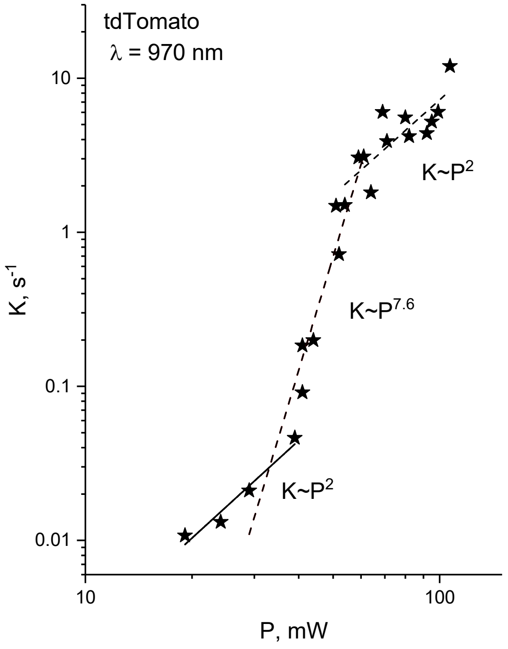

Figure S21. Dependence of bleaching rate K on P for tdTomato at 970 nm in a full power range presented in double logarithmic scale.

12. The dependence of bleaching rate K on P^2 for tdTomato at 1000 nm

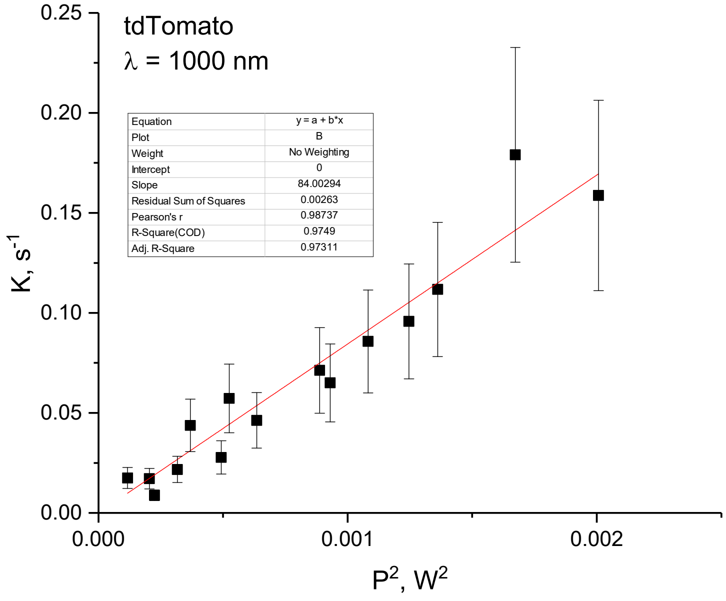

Figure S22. Bleaching rate of tdTomato at 1000 nm in the low power range, presented as a function of P^2. Linear fit is shown by the red line.

13. The dependence of initial (unbleached) fluorescence signal F(0) on P for tdTomato at 1000 nm

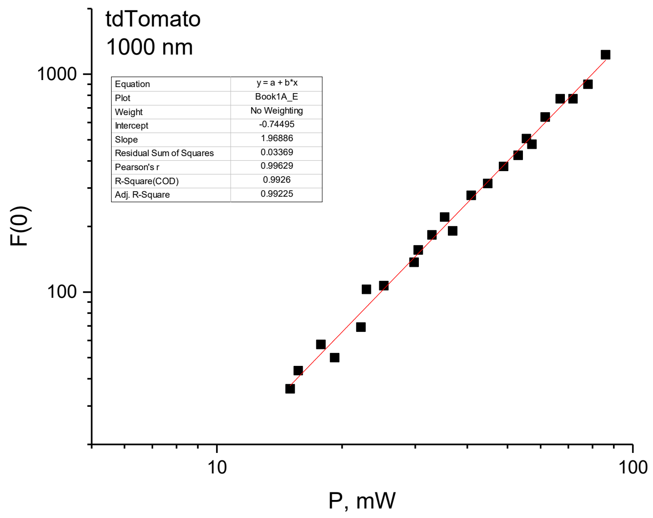

Figure S23. Power dependence of initial (unbleached) fluorescence signal for tdTomato at 1000 nm in double logarithmic scale. The signals are obtained during 100 ms, where the bleaching is negligible. The power exponent, corresponding to the best fit of the dependence (red line) is 1.97 ± 0.04.

14. *The dependence of bleaching rate K on P^3 for tdTomato at 760 nm*

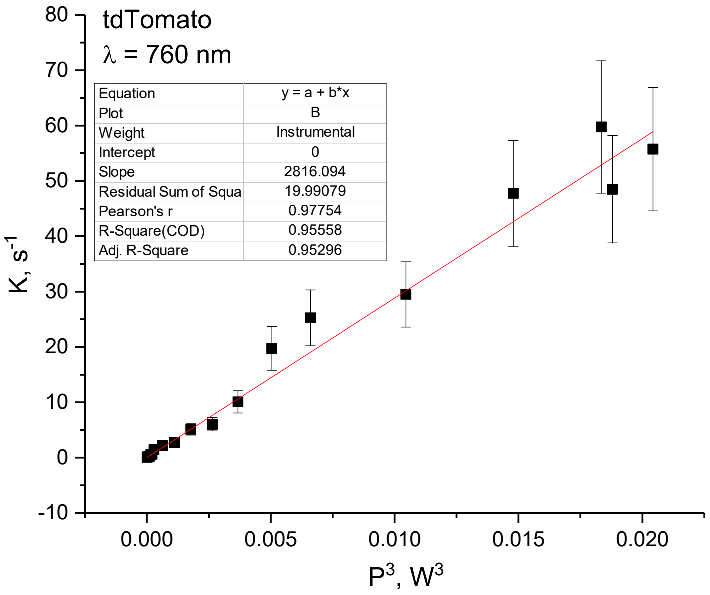

Figure S24. Bleaching rate versus P^3 for tdTomato at 760 nm. The fit corresponding to a saturated 4-th order bleaching, described by eq. (10) with only the main term proportional to P^3, is shown by the red line.

15. *The dependence of bleaching rate K on P for tdTomato at different wavelengths in the K~ P^4 regime with and without saturation*

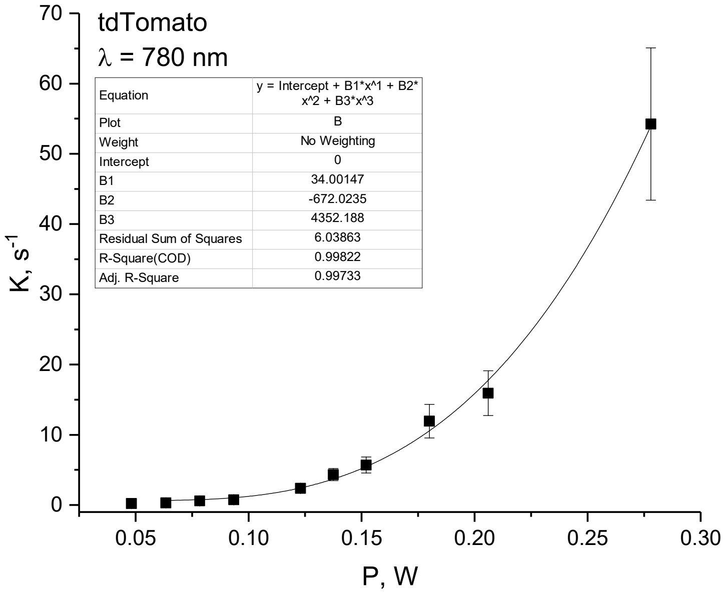

Figure S25. Bleaching rate versus *P* for tdTomato at 780 nm in the region of powers where the $K \sim P^4$ dependence starts to saturate. The fit to a third order polynomial, according to eq. (10), is shown by the red line.

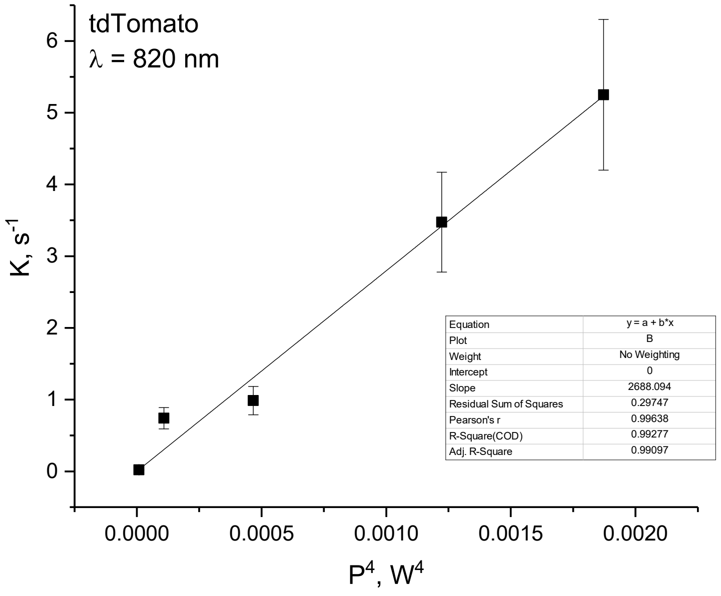

Figure S26. Bleaching rate *K* versus P^4 for tdTomato at 820 nm. The linear fit is shown by the red line.

16. *Laser parameters as a function of wavelength*

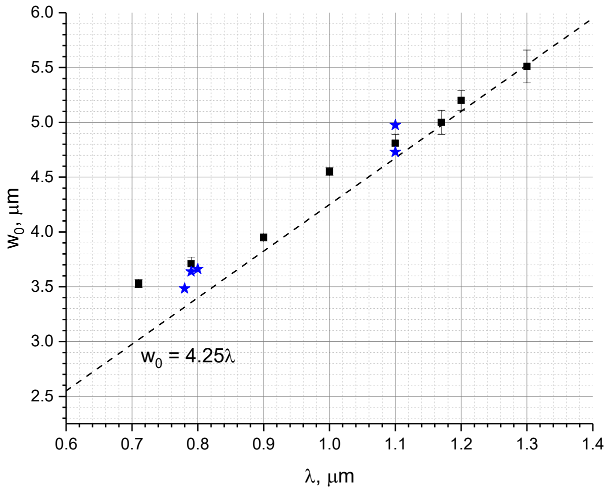

Figure S27. Laser waist w_0 measured after the NA 0.075 objective lens as a function of wavelength. Black squares – razor blade measurements in air, blue asterisks – calculated from the z_R measurement in *E. coli* colonies using convolution method, described in 4.6. Dashed line shows the theoretical prediction for the full back aperture filling, $w_0 = \lambda/\pi NA$.

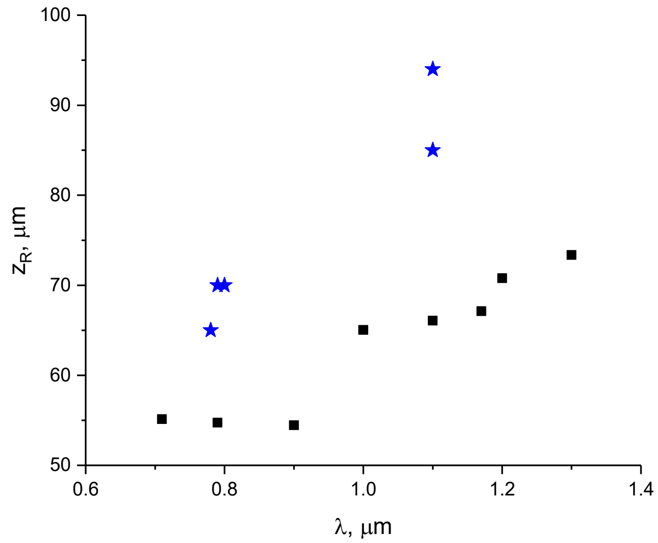

Figure S28. Laser Rayleigh length, z_R, measured after the NA 0.075 objective lens as a function of wavelength. Black squares – razor blade measurements in air, blue asterisks – measurement in *E.*

coli colonies using convolution method, described in 4.6.

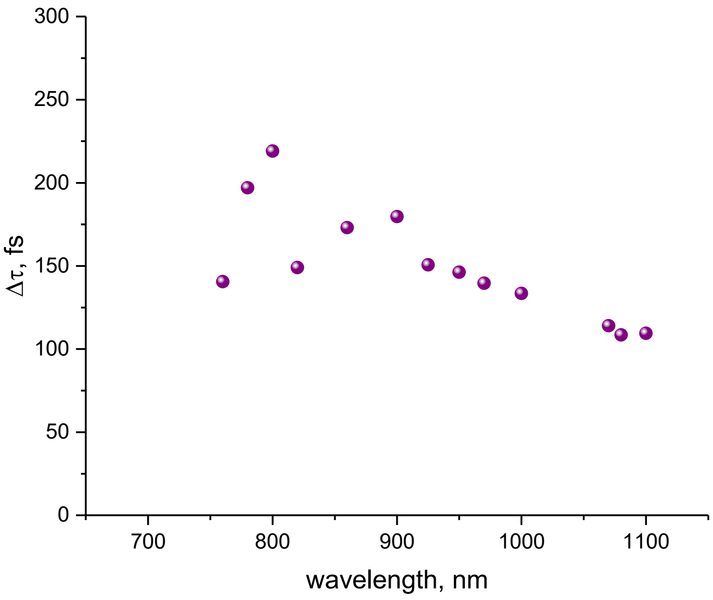

Figure S29. Laser pulse duration as a function of wavelength.

17. Measuring colony thickness and geometrical factor l/2z$_R$*.*

To estimate the thickness of a colony, we measure the dependence of two-photon excited fluorescence from that colony as a function of the z-position (fluorescence z-scan). Some of these z-scans are shown in Figs. S30 – S35. Mathematically the dependence can be described as a convolution of a Lorentzian function with the Rayleigh length z_R, describing the fluorescence intensity squared the focal region and the rectangular function of full width *l*, describing the density of fluorophores inside the colony. Taking the Gaussian-Lorentzian distribution, eq. (2), together with

$$w(z) = \sqrt{1 + (z/z_R)^2}, \qquad \text{(S1)}$$

We can write the convolution integral:

$$F(z) = C \int_{-l/2}^{l/2} \int_0^{\infty} 2\pi r \, I_0^2 \frac{w_0^4}{w^4(z\prime)} \exp\left(-\frac{4r^2}{w^2(z')}\right) dr dz' = \frac{\pi}{4} C I_0^2 \, w_0^2 \int_{-l/2}^{l/2} \frac{dz\prime}{1+(z/z_R)^2}, \qquad \text{(S2)}$$

where $C = \frac{1}{2}\sigma_2 \varphi n$, φ is the fluoresnce quantum yield, *n* is the concentration of fluorophores inside the colony. The result of integration in (S2) depends on the relative values of the colony half-thickness (*l*/2) and Rayleigh length, z_R.

Case (a), $l/2 > z_R$:

$$F(z)/F_0 = \begin{cases} \dfrac{atan\left(\dfrac{lz_R}{z^2+z_R^2-\left(\frac{l}{2}\right)^2}\right)}{\left[\pi+atan\left(\dfrac{lz_R}{z_R^2-\left(\frac{l}{2}\right)^2}\right)\right]}, & at\ |z| > \sqrt{\left(\frac{l}{2}\right)^2 - z_R^2} \\ \dfrac{\left[\pi+atan\left(\dfrac{lz_R}{z^2+z_R^2-\left(\frac{l}{2}\right)^2}\right)\right]}{\left[\pi+atan\left(\dfrac{lz_R}{z_R^2-\left(\frac{l}{2}\right)^2}\right)\right]}, & at\ \ |z| < \sqrt{\left(\frac{l}{2}\right)^2 - z_R^2} \end{cases} \tag{S3}$$

Here $z = 0$ corresponds to a position of focal plane and F_0 is the fluorescence signal at z = 0.

Case (b), $l/2 < z_R$:

$$F(z)/F_0 = \frac{atan\left(\dfrac{lz_R}{z^2+z_R^2-\left(\frac{l}{2}\right)^2}\right)}{atan\left(\dfrac{lz_R}{z_R^2-\left(\frac{l}{2}\right)^2}\right)} \tag{S4}$$

The results of fitting experimental data to functions (S3) (mCherry, jREX-GECO1) and (S4) (tdTomato) are shown in Figs. S30 – S35. Table S1 summarizes the best fitting parameters.

Table S1.

Protein	Colony#	λ, nm	*l*, μm	z_R, μm	$l/2z_R$
mCherry	1	800	166	70	1.19
mCherry	2	1100	240	85	1.41
mCherry	3	1100	196	94	1.04
mCherry	4	790	150	70	1.07
jREX-GECO1	1	780	150	65	1.15
tdTomato	1	1100	160	135	0.59

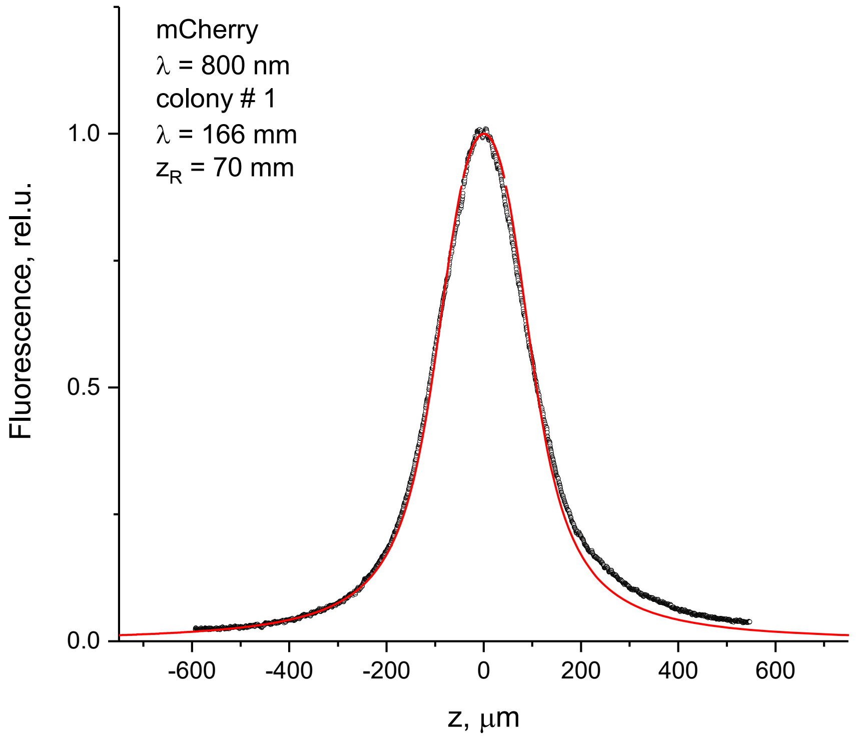

Figure S30. Fluorescence z-scan of a colony expressing mCherry at λ = 800 nm. Symbols – experimental data, red line – fit with function (S3).

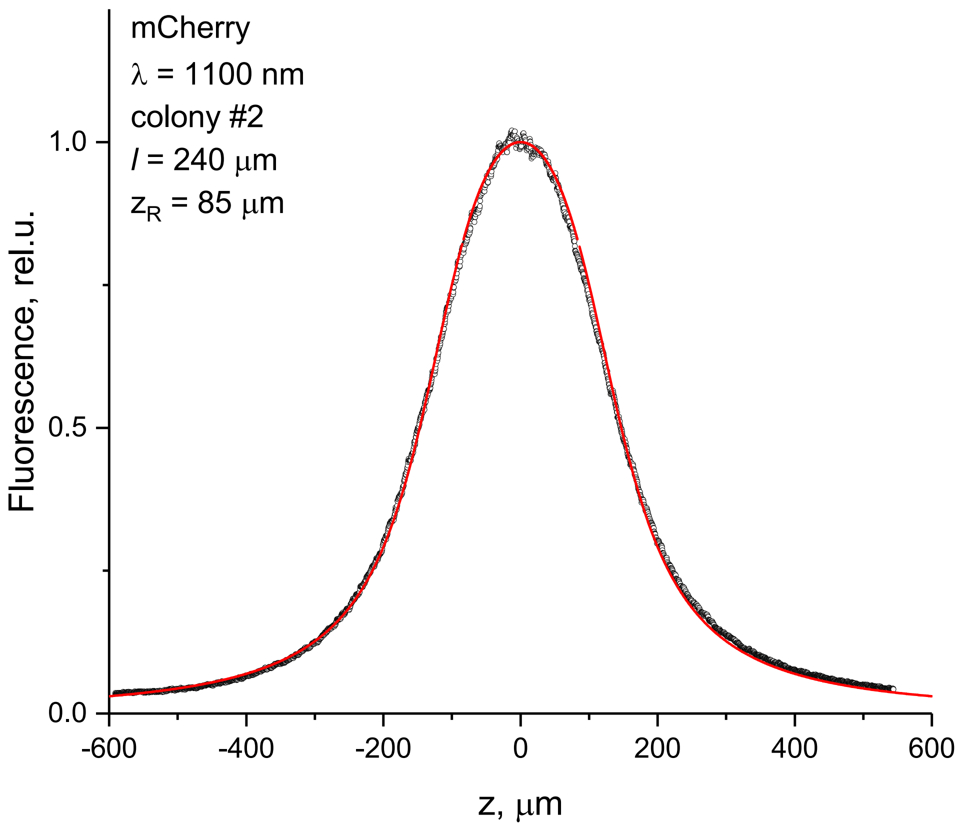

Figure S31. Fluorescence z-scan of a colony expressing mCherry at λ = 1100 nm. Symbols – experimental data, red line – fit with function (S3).

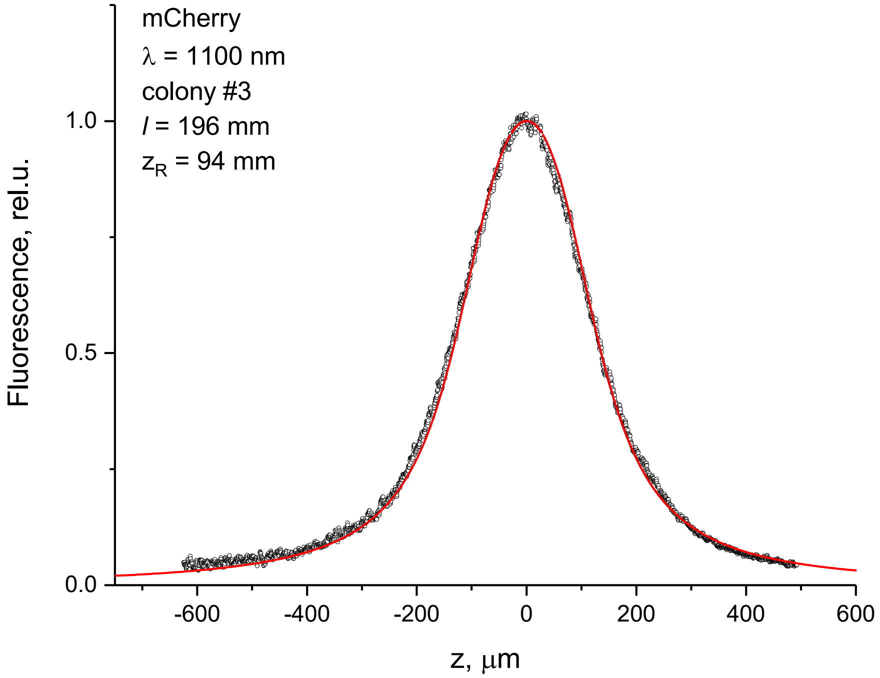

Figure. S32. Fluorescence z-scan of a colony expressing mCherry at λ = 1100 nm. Symbols – experimental data, red line – fit with function (S3).

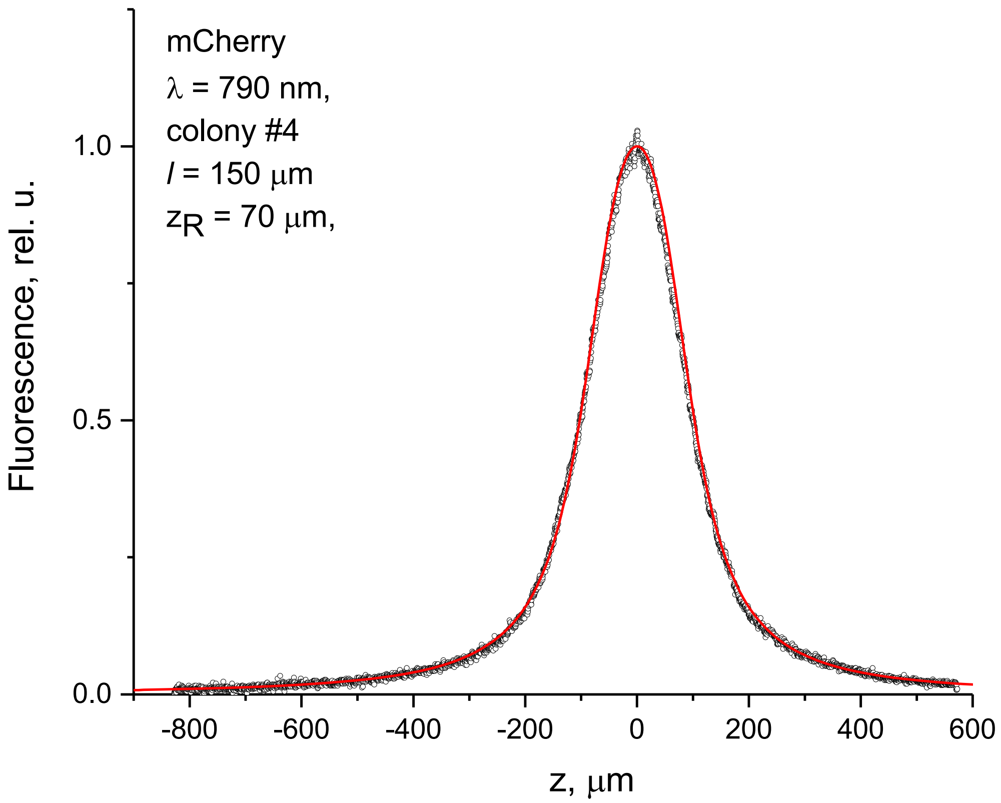

Figure S33. Fluorescence z-scan of a colony expressing mCherry at λ = 790 nm. Symbols – experimental data, red line – fit with function (S3).

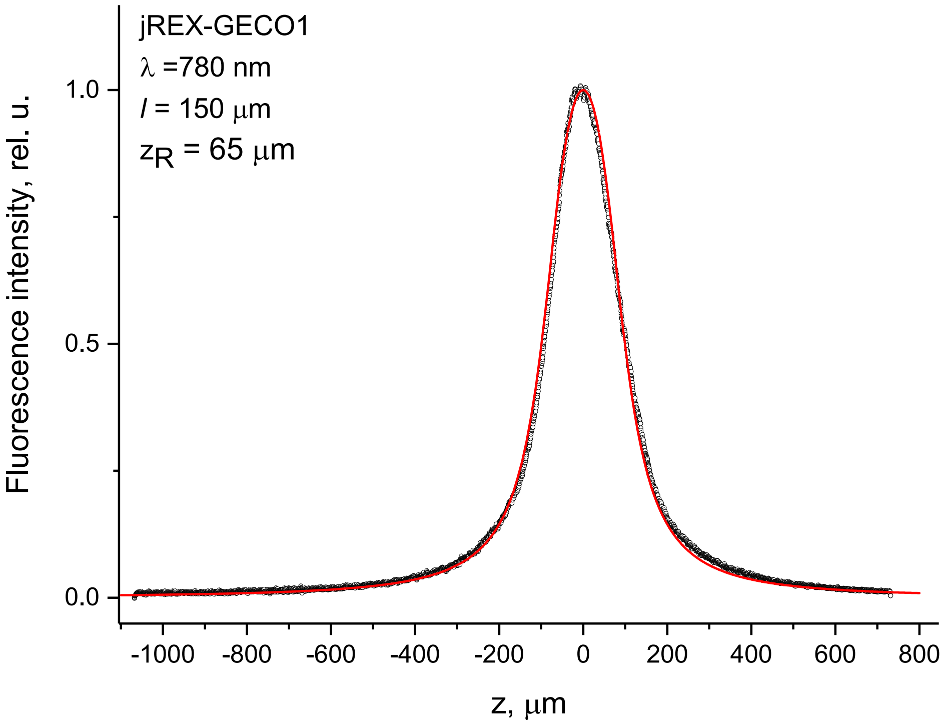

Figure S34. Fluorescence z-scan of a colony expressing jREX-GECO1 at λ = 780 nm. Symbols – experimental data, red line – fit with function (S3).

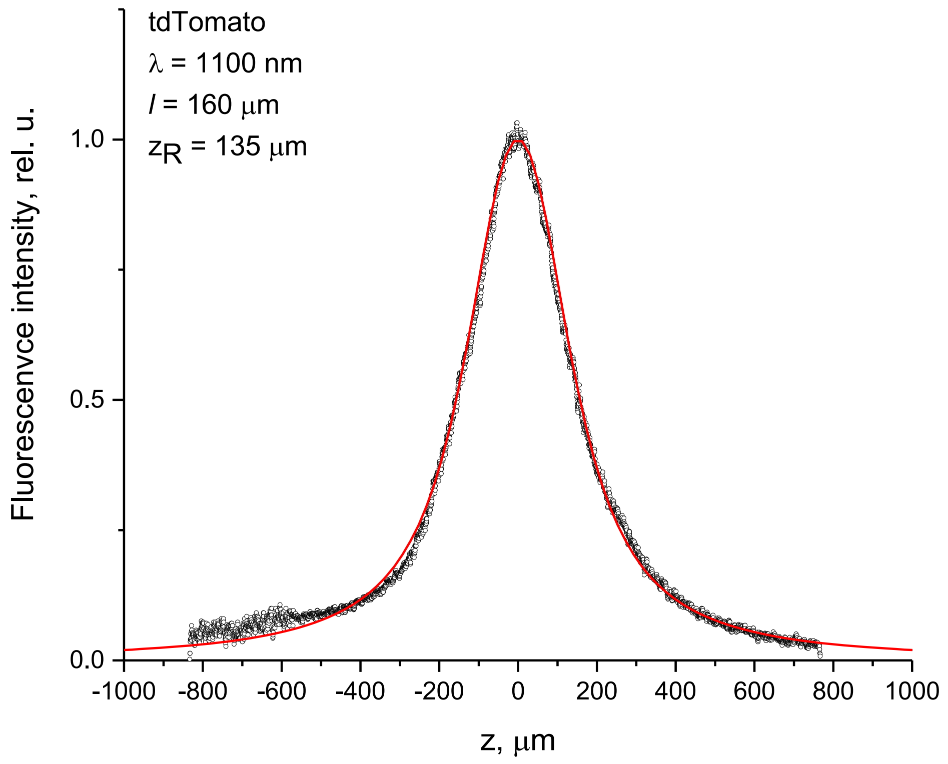

Figure S35. Fluorescence z-scan of a colony expressing tdTomato at λ = 1100 nm. Symbols – experimental data, red line – fit with function (S4).

18. Quantitative comparison of different proteins at different wavelengths for TPLM performance

In the super-quadratic photobleaching regime, the bleaching rate increases with laser intensity faster than fluorescence signal, i.e., P^3-P^4 *vs* P^2. Therefore, it is intuitively clear that augmenting the intensity helps getting brighter image in TPLM only up to a certain level, where very fast bleaching starts to take over. One can ask a practical question: Regarding strongly nonlinear bleaching is there an optimum laser power to provide the maximum photon outcome for a given pixel dwell time? Assuming for simplicity the mono-exponential photobleaching kinetics with the rate K, one can show that the total number of photons emitted from the sample during dwell time t_d is proportional to

$$S = F(0)\frac{1-\exp(-Kt_d)}{K}, \tag{S5}$$

where $F(0)$ is the initial fluorescence intensity. Consider first cubic intensity dependence of the rate (eq. (6) of section 2.1). Then $K = \alpha P^3$. Introducing dimensionless intensity $p = (\alpha t_d)^{1/3}P$ and noticing that $F(0) = Dp^2$, where

$$D = \frac{\varphi_F \sigma_2}{(\alpha t_d)^{2/3}} \tag{S6}$$

is a constant independent on intensity, we re-write (S5) as follows:

$$S = Dt_d \frac{1 - \exp(-p^3)}{p} \quad . \tag{S7}$$

Function f = (1-exp(-p^3))/p has a maximum, see Fig. S36, at p^* = 1.24.

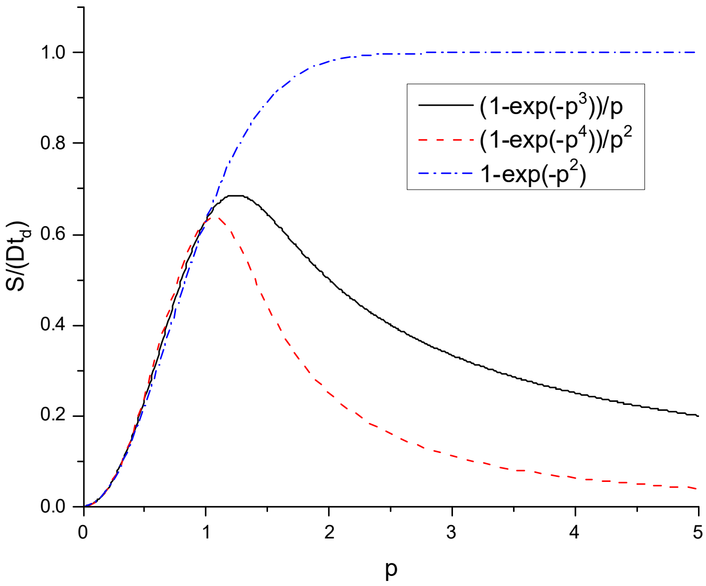

Figure S36. Plots of model functions S7 (black continuous line), S9 (red dashed line), and S11 (blue dash-dotted line).

This corresponds to an optimum laser intensity value *P*, which can be calculated directly if α and t_d are known. In the case of 3PA process without saturation, $\alpha \propto \sigma_2 \sigma_{mn} \varphi_n$, where we only include the molecular parameters (but not the laser parameters and other constants). It is clear that the larger the *D*, the more photons are collected for the dwell time t_d. Substituting the above expression for α into (S6) we obtain molecular "figure of merit" (FOM) for the case of 3PA-induced photochemistry (without saturation):

$$FOM^{(3)} = \frac{\varphi_F \sigma_2^{1/3}}{(\sigma_{mn}\varphi_n)^{2/3}} \tag{S8}$$

The larger this number is (for a molecule undergoing 3PA photochemistry with optimized power according to Fig. S36), the more photons will be collected for a given pixel dwell time.

Consider now the case of non-saturated 4-photon ionization observed for mFruits. It is easy to show that in the case of $K = \beta P^4$,

$$S = Dt_d \frac{1-exp(-p^4)}{p^2}, \tag{S9}$$

where dimensionless power $p = (\beta t_d)^{1/4}P$, $D = \frac{\varphi_F \sigma_2}{(\beta t_d)^{1/2}}$, and $\beta \propto \sigma_2 \sigma_{mn} \sigma_{nc}$. Therefore, the resulting molecular figure of merit is

$$FOM^{(4)} = \frac{\varphi_F \sigma_2^{1/2}}{(\sigma_{mn}\sigma_{nc})^{1/2}} \quad \text{(S10)}$$

Using the last equation, we can directly compare mFruits series since we have all the parameters measured and the mechanism of the bleaching and experimental conditions are the same. The corresponding $FOM^{(4)}$ numbers are collected in Table 5 of the main text.

Finally, in the case of quadratic power dependence of the bleaching rate, $K = \gamma P^2$, we will have:

$$S = Dt_d(1 - exp(-p^2)), \quad \text{(S11)}$$

where $p = (\gamma t_d)^{1/2}P$, $D = \frac{\varphi_F \sigma_2}{\gamma t_d}$, and $\gamma \propto \sigma_2 \varphi_{1,m}$. Function (S11) does not show a maximum, but saturates with increasing power (see Fig. S36). This is because both fluorescence and bleaching rate depend quadratically on power. In this case there is no optimum power, but there is no need to keep p higher than ~2. The molecular figure of merit in this case is equal to

$$FOM^{(2)} = \frac{\varphi_F}{\varphi_{1,m}}. \quad \text{(S12)}$$

The $FOM^{(2)}$ values for all 4 proteins (in the ranges of power where quadratic regime is observed) are presented in Table 6 of the main text.

19. Amino acids responsible for the changes in electrostatic potential at the chromophore of RFPs.

```
               ----0--------10--------20--------30--------40--------50-----
dTomato        MVSKGE----EVIKEFMRFKVRMEGSMNGHEFEIEGEGEGRPYEGTQTAKLKVTKGGPLP  55
mCherry        MVSKGEEDNMAIIKEFMRFKVHMEGSVNGHEFEIEGEGEGRPYEGTQTAKLKVTKGGPLP  55
mPlum          MVSKGE----EVIKEFMRFKEHMEGSVNGHEFEIEGEGEGRPYEGTQTARLKVTKGGPLP  55
jREX-GECO1     LVSKGEEDNMAIIKEFMRFKVHMEGSVNGHEFEIEGEGEGRPYEAFQTAKLKVTKGGPLP

               ---60--------70--------80--------90-------100-------110-----
dTomato        FAWDILSPQFMYGSKAYVKHPADIPDYKKLSFPEGFKWERVMNFEDGGLVTVTQDSSLQD 115
mCherry        FAWDILSPQFMYGSKAYVKHPADIPDYLKLSFPEGFKWERVMNFEDGGVVTVTQDSSLQD 115
mPlum          FAWDILSPQIMYGSKAYVKHPADIPDYLKLSFPEGFKWERVMNFEDGGVVTVTQDSSLQD 115
jREX-GECO1     FAWDILSLQFMYGSKAYIKHPADIPDYFKLSFPEGFRWERVMIFEDGGIIHVNQDSSLQD

               --120-------130-------140-------150-------160-------170-----
dTomato        GTLIYKVKMRGTNFPPDGPVMQKKTMGWEASTERLYPRDGVLKGEIHQALKLKDGGHYLV 175
mCherry        GEFIYKVKLRGTNFPSDGPVMQKKTMGWEASSERMYPEDGALKGEIKQRLKLKDGGHYDA 175
mPlum          GEFIYKVKVRGTNFPSDGPVMQKKTMGWEASSERMYPEDGALKGEMKMRLRLKDGGHYDA 175
jREX-GECO1     GVFIYKVKLRGTNFPPDGPVMQKKTMGWE---EWMYPEDGALKSVIKEGLRLKDGGHYAA

               --180-------190-------200-------210-------220-------230---
dTomato        EFKTIYMAKKPVQLPGYYYVDTKLDITSHNEDYTIVEQYERSEGRHHLFLYGMDELYK   233
mCherry        EVKTTYKAKKPVQLPGAYNVNIKLDITSHNEDYTIVEQYERAEGRHST---GMDELYK   233
mPlum          EVKTTYMAKKPVQLPGAYKTDIKLDITSHNEDYTIVEQYERAEGRHST---GA-----   233
jREX-GECO1     EVRTTYKAKKPVQLPGAYIVDIKLDIVSHNEDYTIVEQCERAEGRH--PTGGMVGLYK   233
```

Fig S37. Alignment of amino acid sequences of RFPS under study. Numeration as in tdTomato

Table S2. Important charges in the chromophore the environment of RFPs That change the electrostatic potential when going from one mutant to another.

Protein	83	163	215
mCherry (d, Å)	0 (11.5)	0 (6.4)	0 (4.4)
DsRed	+1	+1	-1
tdTomato	+1	0	-1
mPlum	0	0	0
jREX-GECO1	0	-1	-1 (?)

d is the distance of the geometrical center of charge from the center of chromophore.
